# Colorectal Cancer Stem Cell States Uncovered by Simultaneous Single‐Cell Analysis of Transcriptome and Telomeres

**DOI:** 10.1002/advs.202004320

**Published:** 2021-02-08

**Authors:** Hua Wang, Peng Gong, Tong Chen, Shan Gao, Zhenfeng Wu, Xiaodong Wang, Jie Li, Sadie L. Marjani, José Costa, Sherman M. Weissman, Feng Qi, Xinghua Pan, Lin Liu

**Affiliations:** ^1^ State Key Laboratory of Medicinal Chemical Biology Nankai University Tianjin 300350 China; ^2^ Department of Cell Biology and Genetics College of Life Sciences The Key Laboratory of Bioactive Materials, Ministry of Education Nankai University Tianjin 300071 China; ^3^ Department of Genetics Yale School of Medicine New Haven CT 06520 USA; ^4^ EHBIO Gene Technology co., LTD Beijing 100029 China; ^5^ School of Mathematical Sciences Nankai University Tianjin 300071 China; ^6^ Department of General Surgery Tianjin Medical University General Hospital Tianjin 300052 China; ^7^ Department of Biology Central Connecticut State University New Britain CT 06050 USA; ^8^ Department of Pathology, Yale School of Medicine New Haven CT 06520 USA; ^9^ Department of Biochemistry and Molecular Biology School of Basic Medical Sciences Southern Medical University Guangzhou Guangdong Province 510515 China; ^10^ Guangdong Provincial Key Laboratory for Single Cell Technology and Application Guangzhou Guangdong Province 510515 China; ^11^ Institute of Translational Medicine Tianjin Union Medical Center Nankai University Tianjin 300000 China

**Keywords:** cancer stem cells, colorectal cancer, epithelial cells, heterogeneity, single‐cell RNA‐seq, telomere length

## Abstract

Cancer stem cells (CSCs) presumably contribute to tumor progression and drug resistance, yet their definitive features have remained elusive. Here, simultaneous measurement of telomere length and transcriptome in the same cells enables systematic assessment of CSCs in primary colorectal cancer (CRC). The in‐depth transcriptome profiled by SMART‐seq2 is independently validated by high‐throughput scRNA‐seq using 10 × Genomics. It is found that rare CSCs exist in dormant state and display plasticity toward cancer epithelial cells (EPCs) that essentially are presumptive tumor‐initiating cells (TICs), while both retaining the prominent signaling pathways including WNT, TGF‐*β*, and HIPPO/YAP. Moreover, CSCs exhibit chromosome copy number variation (CNV) pattern resembling cancer EPCs but distinct from normal stem cells, suggesting the phylogenetic relationship between CSCs and cancer EPCs. Notably, CSCs maintain shorter telomeres and possess minimal telomerase activity consistent with their nonproliferative nature, unlike cancer EPCs. Additionally, the specific signature of CSCs particularly *NOTUM*, *SMOC2*, *BAMBI*, *PHLDA1*, and *TNFRSF19* correlates with the prognosis of CRC. These findings characterize the heterogeneity of CSCs and their linkage to cancer EPCs/TICs, some of which are conventionally regarded as CSCs.

## Graphical abstract

Cells enriched from colorectal cancer with canonical CSC markers CD44, CD133 and LGR5 contain not only CSCs, but also a variety of other cell types, particularly EPC_A and B. Single cell multiomics of transcriptome and telomere reveals the phylogenetic relationship of cancer EPCs with, and specific characters of CSCs. These results are validated by ImmunoFISH, CRISPR/Cas9 knockout and other analyses, and uncover the hidden heterogeneity of colorectal CSCs.

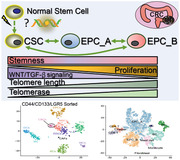

## 1 Introduction

A subset of cancer cells named as cancer stem cells (CSCs) presumably are capable of self‐renewal to initiate and maintain tumor growth, contribute to tumor recurrence and are resistant to conventional chemotherapy.^[^ ^1^ ^]^ It also evokes the attractive possibility of elimination of malignant tumors by defining and targeting the critical requirements of CSC population.^[^ ^2^ ^]^ CSCs in most human tumors, including colorectal cancer (CRC), traditionally are identified by cell surface markers, such as CD133/PROM1,^[^ ^3^ ^]^ CD44,^[^ ^4^ ^]^ or LGR5.^[^ ^5^ ^]^ Yet, stem cell hierarchy may be much more heterogeneous than previously appreciated, complicating the identification and eradication of CSCs.^[^ ^1^, ^6^ ^]^ Identification of CSC populations based on current bulk cell analyses could miss subsets of residual CSCs that are typically rare and not easily isolated.^[^ ^7^ ^]^ Thus far, various definitions about CSCs, CSC‐like cells, or tumor‐initiating cells (TICs) have been proposed in various tumors.^[^ ^3^, ^8^ ^]^ Hence, tumors may contain a more complicated composition of CSC‐like cells or precursor cells that contribute to their tumor‐propagating potential than previously thought.

Single‐cell transcriptome techniques enable unbiased characterization of the cellular diversity of tissues and allow identification of distinct cell subtypes in cancer, including CSCs.^[^ ^1^, ^7^, ^9^ ^]^ For instance, single‐cell analysis uncovered that CSCs fuel the growth of oligodendrogliomas, and that these CSCs show signs of proliferation, while other cancer cells do not.^[^ ^9^ ^]^ A small number of CSCs in leukemia are highly resistant to treatment and are likely responsible for disease recurrence when the treatment is stopped.^[^ ^7^ ^]^ Single‐cell quantitative polymerase chain reaction (qPCR),^[^ ^10^ ^]^ and single‐cell RNA‐sequencing (scRNA‐seq) have revealed the heterogeneity and distinct subpopulations within human CRC.^[^ ^11^ ^]^ Moreover, single‐cell analyses by RNA sequencing, methylation profiling and mutation characterization provide further insights into intratumoral heterogeneity and the epigenetic dynamics of human CRCs.^[^ ^12^ ^]^

Telomeres are highly repetitive ribonucleoprotein structures that protect chromosome ends and maintain genomic stability, essential for cell proliferation and immortalization.^[^ ^13^ ^]^ Dysregulation of telomeres and telomerase, the enzyme responsible for maintaining telomeres, results in aging as well as cancer.^[^ ^13^ ^]^ Intriguingly, while telomere shortening and heterogeneity are found in CRC and other tumors,^[^ ^14^ ^]^ long telomeres are claimed to be essential for proliferation of cancer cells.^[^ ^13^, ^15^ ^]^ It is likely that telomere analysis based on bulk cell samples may not be able to distinguish various subcell types in a heterogeneous tumor tissue. Hence it remains elusive whether CSCs have long or short telomeres, and whether they are proliferative or quiescent. The transcriptome features together with the telomere status of cell subpopulations and particularly potential CSCs in human CRC have not been characterized at single‐cell level.

Single‐cell telomere length measurement by qPCR^[^ ^16^ ^]^ coupled with single‐cell transcriptome analysis in the same cell,^[^ ^17^ ^]^ provide new opportunity to characterize specific global transcription profile and the telomere length in the individual cell. By taking advantage of simultaneous analysis of the transcriptome and telomere length in the same cell, we investigated the association of telomere length and transcriptome in the same cell of primary CRC tumors following enrichment of CSCs by fluorescence‐activated cell sorting (FACS) using known cell surface markers for “CSCs,” and independently validated the outcome by scRNA‐seq on 10 × Genomics. We found that in native CRC tissue, CSCs are dormant, are indeed rare, unexpectedly maintain short telomeres, and can be characterized by high expression of specific genes. Furthermore, the CSCs can be transformed to cancer EPCs that express telomerase and acquire longer telomeres, which are required for tumor proliferation.

## 2 Results

### 2.1 Profiling of Human Primary CRC at Single‐Cell Level by Integrated Analysis of Telomere Length and Transcriptome

Freshly resected primary tumors from eight CRC patients who were treatment‐naïve at the time of surgery (Table S1, Supporting Information), were dissociated into single cells. The single cells were processed for single‐cell analysis of transcriptome and telomere length in the same cell based on the single‐cell telomere length and transcriptome sequencing method (scT&R‐seq) (Figure S1a, Supporting Information).^[^ ^17^ ^]^ FACS by several commonly known surface markers for CSCs in CRC was employed to enrich potential CSCs. Dead cells initially were excluded by 7‐AAD^+^ (Figure S1b, Supporting Information) prior to sorting potential CSCs by canonical and widely reported CSC markers of CRCs, including CD44, CD133/PROM1, and LGR5 (Figure S1c, Supporting Information). As sphere formation can also enrich potential CSCs,^[^ ^18^ ^]^ the cells isolated from sphere formation (Sphere) and their progenitor suspensions (Adhere) were also included for single‐cell analysis (Figure S1d, Supporting Information). After RNA‐seq quality control (QC) filtration of all 831 individual cells sequenced (Figure S1d, Supporting Information and see the Experimental Section), 693 individual cells were retained with high quality scRNA‐seq data for subsequent analysis (Figure S1d, Supporting Information). Approximately 600 000 paired‐end mapped reads on average were obtained from each cell (Figure S2a,b, Supporting Information). The average number of genes expressed in each cell was about 4100 (Figure S2c,d, Supporting Information). The number of transcripts in each cell from different patients (batch) were similar (Figure S2b,d, Supporting Information). Major cell types in CRCs were classified based on their transcriptome profile.

To explore the cellular composition of CRCs, we applied principal component analysis (PCA) on variably expressed genes across all cells by *Seurat* (Figure S2e, Supporting Information).^[^ ^19^ ^]^ The top 20 significant principal components were used for cluster analysis (Figure S2f, Supporting Information). We did not observe batch effects in these clusters, including raw sequencing depth (Figure S2g, Supporting Information), human genome mapping ratio (hg38) (Figure S2h, Supporting Information) and CRC patient samples (Figure S2i, Supporting Information), indicating minimal biases due to potential batch effects, and thus ensure the classifications were appropriate.

### 2.2 Single‐Cell Profiling Distinguishes CSCs from Non‐CSCs in CRC

**Figure 1:**
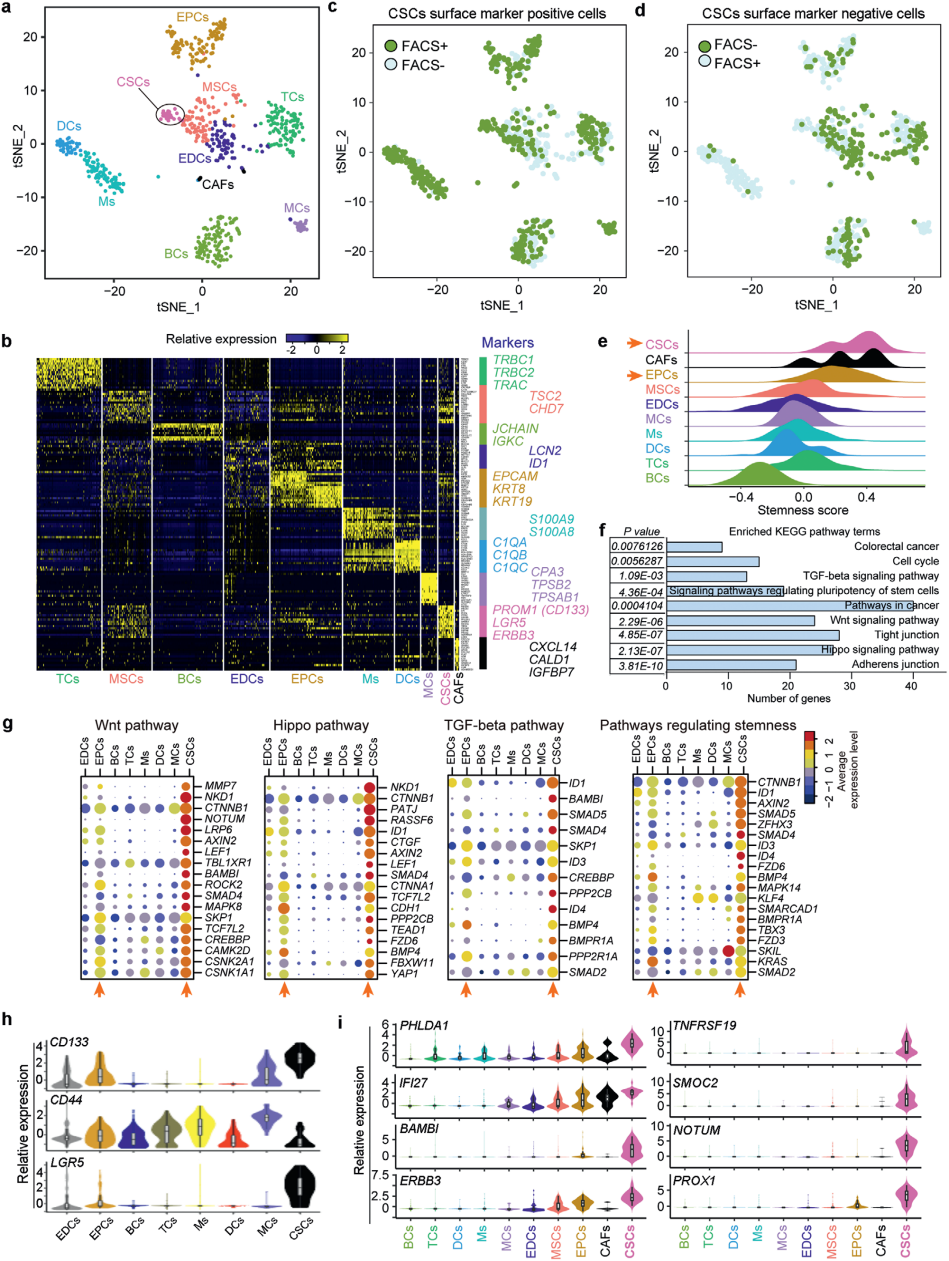
Classification of subpopulations in primary CRC tumors and molecular signature of CSCs delineated by single‐cell transcriptome analysis. a) Unsupervised graph‐based clustering of single cell RNA‐seq dataset projected onto a t‐SNE plot displaying distinct subpopulations of CRC tumors. A total of 693 cells were clustered into 10 distinct groups. Each point represents an individual cell. Cell clusters were labeled and colored by subcell type names: TCs, T cells; MSCs, mesenchymal cells; BCs, B cells; EDCs, endothelial cells; EPCs, epithelial cells; Ms, macrophages; DCs, dendritic cells; MCs, mast cells; CSCs, cancer stem cells; CAFs, cancer‐associated fibroblasts. b) Heatmap of top representative marker genes for each cluster identified by Seurat. Top marker genes were determined by ROC test (see the Experimental Section). Gene expression profiles of selected marker genes were used to assign cell classification. Columns represent individual cells; rows represent genes. Gene expression clusters were generated in Seurat using scaled normalization data. Identified cluster names are indicated at the bottom. c) t‐SNE plot showing distribution of cells sorted by known cell surface CSC positive markers, including CD44, CD133/PROM1 and LGR5. 693 single cells are plotted. Each point represents a cell. The green and blue dots represent FACS^+^ and FACS^−^ cells, respectively. d) t‐SNE plot showing distribution of cells that are negative for markers, CD44, CD133/PROM1, and LGR5. 693 single cells are plotted. Each point represents a cell. The green and blue dots represent FACS^−^ and FACS^+^ cells, respectively. e) Density distribution of stemness score in subpopulations. The “stemness score” signatures were calculated by the average relative expression level of a key stemness related gene set, previously reported and validated.^[^ ^9^ ^]^ f) KEGG enrichment of highly expressed genes in CSCs with AUC>0.8. Selected 8 pathways ranked by *P*‐value are shown. g) Expression of key markers and signaling pathways of CSCs. Dot plots of functionally relevant genes involved in WNT pathway, HIPPO pathway, TGF‐*β* pathway and stemness from KEGG enrichment analysis. *CTNNB1* also known as *β‐Catenin*. The color key from navy to red indicates low to high average gene expression level, respectively. The dot size indicates percentage of cells expressing a certain marker. h) Violin plot showing expression distribution of known commonly used CSC marker genes (*PROM1*, *CD44*, *LGR5*) in each subpopulation. i) Violin plot of specifically enriched genes for CSCs. These genes show high level of Area Under the Curve values (AUC > 0.9). For each gene, the AUC value (ROC test) corresponds to the “classification power.” AUC values ranging from 0 for random, to 1 for perfect classifier for a given cluster. Cells are color‐coded by subpopulations.

By reducing the dimensionality of the data using t‐SNE (t‐distributed stochastic neighbor embedding), we identified 10 distinct clusters (**Figure** **1**a), including T cells (TCs), B cells (BCs), mesenchymal cells (MSCs), endothelial cells (EDCs), epithelial cells (EPCs), macrophages (Ms), dendritic cells (DCs), mast cells (MCs), CSCs, and cancer‐associated fibroblasts (CAFs). These 10 distinct subpopulations were further systematically defined based on the canonical markers for distinct cell types (Figure 1b; Tables S2 and S3, Supporting Information). In addition, gene ontology (GO) analysis of highly expressed genes in each subpopulation (*P* < 0.01, Table S2, Supporting Information), also matched the tissue‐specific functions of each cell type (Table S4, Supporting Information). For example, the top GO enrichment (Biological Process) hits of BCs include B cell activation, leukocyte differentiation, and adaptive immune response. Top GO hits of EPCs include morphogenesis of a polarized epithelium and EPC development. Examination of canonical marker genes also revealed major cell populations when plotted on the t‐SNE plot (Figure S3a, Supporting Information), further demonstrating the accuracy of clustering and robustness of our data.

Indeed, cells in the CSC cluster highly expressed known CSC markers *PROM1*/*CD133* and *LGR5*, as well as the stemness related gene *ERBB3*, and *SOX* family genes (*SOX4*, *SOX6*, and *SOX9*) (Figure 1b). The crypt stem cell marker LGR5 also marks a subpopulation of adenoma cells that fuel the growth of established intestinal adenomas by lineage retracing.^[^ ^20^ ^]^ The normal intestinal stem cell marker genes *BMI1* ^[^ ^21^ ^]^ and *LRIG1* ^[^ ^22^ ^]^ were also highly expressed in CSCs and EPCs (Figure S3b, Supporting Information). In addition, we observed rare CAFs in the niche (Figure 1a), which can promote cancer formation and chemo‐resistance by sustaining cancer stemness.^[^ ^23^ ^]^ Furthermore, previously identified CAF markers, *CXCL14*,^[^ ^24^ ^]^ *CALD1*,^[^ ^25^ ^]^ and *IGFBP7*,^[^ ^25^ ^]^ were highly expressed in CAFs also based on our RNA‐seq data (Figure 1b).

### 2.3 New Feature of CRC CSCs Sorted by Known CSC Markers

To efficiently capture and distinguish potential features of CSCs from non‐CSCs in primary tumors, we pooled all cells from maker positive and negative populations for cell cluster analysis (Figure S1d, Supporting Information). Of these gated cells following FACS isolation, CSC positive markers enriched more CSCs, while CSC marker negative cells enriched more TCs, BCs, and EDCs (Figure 1c,d). CSCs mostly were enriched from CD44+CD133+ and CD44−CD133+ sorted cell population (Figure S1d, Supporting Information). However, cells from the spheres only clustered to EPCs and EDCs, and none to CSCs (Figure S1d, Supporting Information). These results are also supported by the findings that tumorspheres can be derived from both cancer cell lines^[^ ^26^ ^]^ and nonstem cells (such as stem cell transit‐amplifying progeny) of cancer tissues, whereas normal quiescent stem cells fail to form tumor spheres.^[^ ^18^ ^]^

It is worth noting that even though we employed FACS and sorted the cells dissociated from the primary CRC tumors to enrich CSCs by commonly used CSC surface markers, the proportion of CSCs was still low (≈3.9%) in the whole cell population, and LGR5+ cells were still heterogeneous (Figure S1d, Supporting Information), further highlighting the importance and necessity of single‐cell analysis, which presumably can capture the unique features of rare CSCs in a tumor.

### 2.4 Signaling Pathways and Marker Genes of CSCs in CRC

To facilitate the quantitative evaluation of stemness in each cell type, we defined the “stemness score”^[^ ^9^ ^]^ across all single cells, calculated as the average relative expression of solid cancer stemness‐related genes (Table S5, Supporting Information), and these stemness‐related genes also were listed in CRC reported early.^[^ ^27^ ^]^ Indeed, CSCs were endowed with high stemness score signatures, and interestingly EPCs also exhibited stemness signatures but to a lesser extent. In contrast, terminally differentiated B‐cells (BCs) displayed the lowest stemness score (Figure 1e).

Furthermore, we performed Kyoto Encyclopedia of Genes and Genomes (KEGG) pathway enrichment analysis to characterize the distinct pathways enriched in CSCs (Table S6, Supporting Information). The top enriched terms included signaling pathways regulating pluripotency of stem cells as expected, as well as adherence and tight junctions, canonical WNT, HIPPO, TGF‐*β* signaling pathway, and CRC related pathways (Figure 1f). These pathways play a key role in maintaining the stemness and undifferentiated state of CSCs in CRC, such as epithelial–mesenchymal transition (EMT) linked to cancer progression and metastasis.^[^ ^25^, ^28^ ^]^ Ligand *CTNNB1* (*β‐CATENIN*), effectors *LEF1* and *TCF7L2*, WNT targets *AXIN2* and *MMP7*, WNT receptors such as *LRP5*, *LRP6*, *FZD1*, *FZD3*, and *FZD6* were highly expressed in CSCs (Figure 1g). The WNT signaling pathway regulates the development of stem cells, including CSCs.^[^ ^28b^ ^]^ *LGR5*,^[^ ^5b^ ^]^ *CD44* ^[^ ^29^ ^]^ and *SOX9* ^[^ ^30^ ^]^ are WNT target genes. Canonical WNT/*β*‐CATENIN signaling pathway enables nuclear translocation of *β*‐Catenin and TCF/LEF‐dependent gene transactivation in human intestinal EPCs.^[^ ^28b^ ^]^ In the intestinal epithelium, *β*‐CATENIN and TCF couple proliferation and differentiation to the sorting of cell populations.^[^ ^28c^ ^]^ HIPPO pathway effector *YAP1* and YAP target genes (*TJP1*, *CYR61*, *TEAD2*, *TEAD1*, *CTGF*) were highly expressed in CSCs, in contrast to other subpopulations, except for EPCs (Figure 1g). The TGF‐*β* pathway was strongly activated in CSCs as evidenced by high expression levels of the targets *ID1*, *ID3*, and *ID4*, receptor‐regulated SMADs (*SMAD2*, *SMAD4*, and *SMAD5*), and ligand *BMP4* and receptors (Figure 1g).

Classical CSC marker genes *PROM1/CD133*, *CD44*, and *LGR5* were highly expressed in CSCs isolated from primary CRCs (Figure 1h). Remarkably, EPCs also expressed these common CSC marker genes. Moreover, our single‐cell analysis identified specific genes enriched for CRC CSCs, including *PROX1*, *TNFRSF19/TROY*, *SMOC2*, *NOTUM*, *BAMBI*, *PHLDA1*, *IFI27*, and *ERBB3*, and these genes were expressed at remarkably higher levels in CSCs distinct from any other cell type (Figure 1i). Earlier, cells positive for *Prox1* show stem cell activity in mouse intestinal adenomas.^[^ ^31^ ^]^ *Tnfrsf19* and *Smoc2* also are highly expressed in mouse intestinal stem cells.^[^ ^5^, ^32^ ^]^

These analyses reveal the global gene expression pattern that distinguishes CSCs from niche cells in CRCs. Interestingly, the signaling pathways of the EPC clusters resemble those of CSCs, unlike other nonepithelial cancer cell‐types (Figure 1g).

### 2.5 Linkage and Difference between CSCs and EPCs in CRC

**Figure 2:**
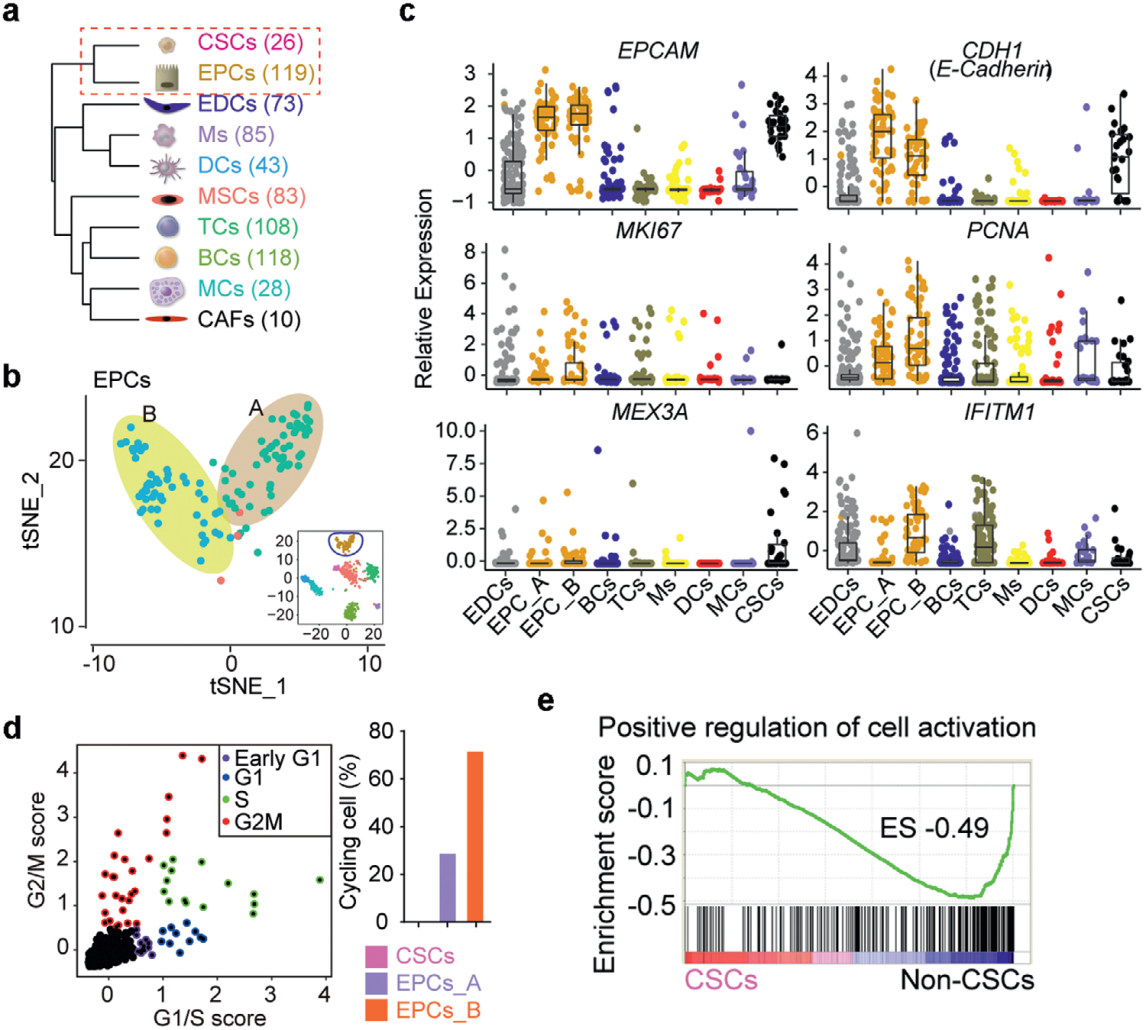
Similarities and differences among CSCs, EPCs_A and EPCs_B. a) Unsupervised hierarchical clustering of the 10 clusters based on the average gene expression of single cells in each subpopulation. Each node represents a subpopulation. Number of cells analyzed for each subpopulation are shown on the right side. b) t‐SNE plot showing distinct subpopulations of EPCs. EPCs could be distributed into two subpopulations under high resolution (t‐SNE resolution = 0.8) by Seurat, termed EPCs_A and EPCs_B. c) Expression of epithelial lineage marker genes (*EPCAM*, *CDH1* also known as *E‐CADHERIN*) and cell proliferation markers (*MKI67* and *PCNA*) in each cell. CSCs and EPCs exhibit similar expression patterns. d) Classification of single cells into cell‐cycle phase based on expression patterns of selected cell‐cycle specific genes.^[^ ^9^ ^]^ Color represents a cycling cell; black represents noncycling. Right panel indicates distribution of cycling cells in EPCs and CSCs. e) GSEA analysis of signature in GO term of “positive regulation of cell activation” in CSCs compared with non‐CSCs. Enrichment score (ES) was calculated by GSEA software from the Broad Institute. Black bars represent individual genes in rank order.

Unsupervised hierarchal clustering analysis showed that EPCs and CSCs were clustered together, distinct from immune cells, indicating that EPCs and CSCs are more closely related than other cells (**Figure** **2**a). Nevertheless, EPCs could be further classified into two subpopulations under high resolution (=0.8) by t‐SNE, which were arbitrarily termed EPCs_A and EPCs_B (Figure 2b; Figure S4a, Supporting Information). A set of 98 up‐regulated genes were shared in CSCs and these two subtypes of EPCs, and associated with the specific functions of cancer EPCs (Figure S4b, Supporting Information). By GO analysis, the most upregulated genes (1826; Table S7, Supporting Information) in EPCs_B were uncovered to participate in cell cycle related terms, such as regulation of mitotic cell cycle and cell cycle G2/M phase transition (*P* < 10^−10^) (Figure S4c,d, Supporting Information). Both EPCs and CSCs expressed epithelial lineage markers *EPCAM* and *CDH1/E‐CADHERIN* at higher levels than did other cell types, suggesting their potential linkage of the cancer EPC types (Figure 2c). Nevertheless, cell proliferation genes including *PCNA* and *MKI67* were expressed at higher levels in EPCs_B compared with EPCs_A, but not or only minimally expressed in CSCs (Figure 2c). While the rare CSCs instead highly expressed *MEX3A* (Figure 2c), *Mex3a* is shown to express in a subset of LGR5^+^ cells that proliferate slowly and MEX3A^+^ cells are multipotent and can generate all mouse intestinal lineages including epithelium.^[^ ^33^ ^]^

Additionally, we analyzed the cell‐cycle status using expression signatures for G1/S and G2/M phase specific genes.^[^ ^9^ ^]^ Consistent with proliferation markers, most of the high cycling score cells belong to EPCs, and the proportion of cycling cells from EPCs_B was higher than that of EPCs_A, in contrast to CSCs (Figure 2d). Further, comparative gene expression analysis and gene set enrichment analysis (GSEA) on CSCs and other subpopulations revealed a noticeable downregulation of a large number of genes associated with the gene ontology (GO) term positive regulation of cell activation (Figure 2e). These data suggest that the CSCs in CRC may be quiescent or dormant and that the EPCs_B subpopulation might represent major proliferative CSCs‐like cells or tumor initiating cells (TICs) in CRC.

### 2.6 scT&R‐seq Analysis Reveals Short Telomeres in CSCs of CRC

**Figure 3:**
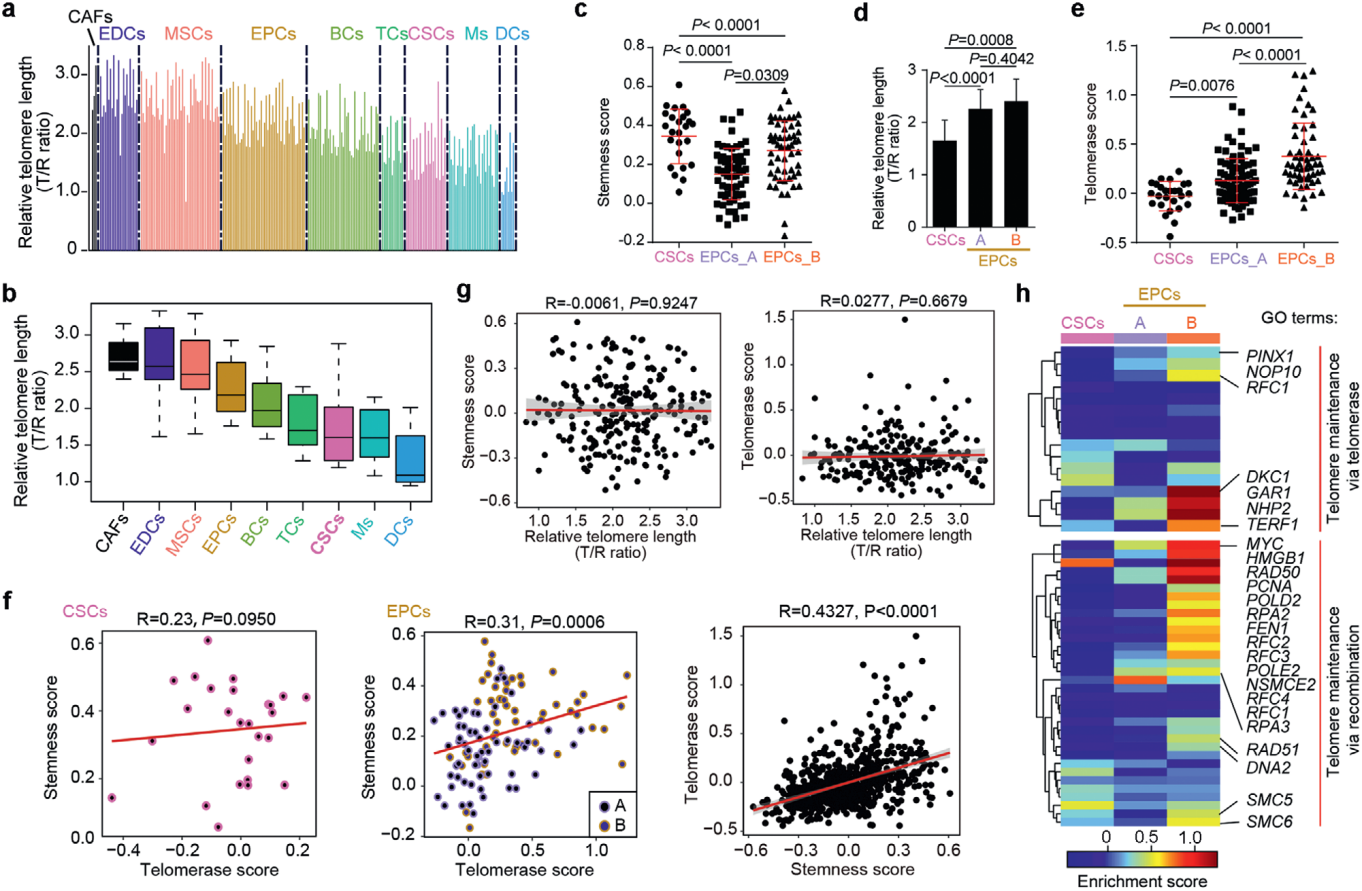
Telomere length and its significance in CSCs and EPCs of CRC tumors. a) Histograms displaying single‐cell telomere length. Relative telomere length shown as T/R ratio was measured by qPCR after preamplification step that simultaneously amplifies both telomere repeats (T) and a reference gene (R). Each histogram represents an individual cell. Cells were grouped and color‐coded by distinct subcell types. b) Boxplot showing distribution of telomere length in subpopulations of CRCs. Each box shows the median and interquartile range (IQR 25th–75th percentiles), and whiskers indicate the highest and lowest value within 1.5 times the IQR. c) Distribution of stemness score in CSC cluster and EPC subclusters. d) Mean telomere length in CSC cluster and EPC subclusters. e) Distribution of predicted telomerase signature in CSC cluster and EPC sub‐clusters. Mean ± SEM, *P*‐values based on one‐way ANOVA. f) Scatter plot illustrating predicted telomerase signature and stemness score in CSCs, EPCs, and all single cells. A linear regression line is also shown. g) Correlation among stemness score, predicted telomerase signature and telomere length. A linear regression line was shown. Shading represents the 95% confidence interval for regression line. h) Heatmap displaying expression pattern of telomere‐related genes in EPCs and CSCs. Two lists of telomere‐related genes were from “GO_telomere_maintenance_via_telomerase” and “GO_telomere_maintenance_via_recombination” in GSEA (http://software.broadinstitute.org/gsea/index.jsp).

Using a well‐established method for simultaneous measurement of telomere length in combination with RNA‐seq analysis (scT&R‐seq) in the same cell,^[^ ^16^, ^17^ ^]^ we obtained both scRNA‐seq and relative telomere length data from 302 single cells (Table S8, Supporting Information). A total of 242 cells passed the RNA‐seq and telomere length QC concurrently (**Figure** **3**a, see the Experimental Section). Relative telomere length, shown as T/R ratio, in CRC tumors at the single‐cell level varied considerably ranging from 0.83 to 3.33 (Figure 3a). Of the subpopulations, EDCs and CAFs had the longest telomeres on average, whereas dendritic cells (DCs) displayed the shortest mean telomeres (Figure 3b). Surprisingly, CSCs contained shorter telomeres compared with most of other cell types (Figure 3b).

Furthermore, we systematically compared the telomere length and features among CSCs, EPCs_A, and EPCs_B. The stemness score significance of EPCs_B resembled more closely CSCs than did EPCs_A, and EPCs_B differed from EPCs_A (*P* = 0.0309) (Figure 3c), again suggesting that EPCs_B may arise from CSCs. Intriguingly, both EPCs_B and EPCs_A cell types had longer telomeres than did CSCs (Figure 3d). The immediate questions were why their telomere lengths differed and how the telomere lengths were maintained in CSCs and EPCs. As telomeres are primarily maintained by telomerase, we then predicted telomerase activity (telomerase score) through a series of gene signatures that were reported and validated in human cancer cell lines^[^ ^14a^ ^]^ (Table S5, Supporting Information). EPCs exhibited a higher “telomerase score” than did other subpopulations, and EPCs_B cells expressed higher telomerase activity than did EPCs_A and CSC cells (Figure 3e). The stemness score of CSCs was dissociated with telomerase activity (Figure 3f, *P* = 0.0950), while the stemness score was significantly correlated with telomerase activity in EPCs (Figure 3f, *P* = 0.0006). When all cells were pooled, the stemness score significantly correlated with telomerase activity (Figure 3f, *P* < 0.0001). Yet, telomere length did not correlate with stemness score (Figure 3g, *P* = 0.9247), nor with telomerase activity in the pooled cells (Figure 3g, *P* = 0.6697), likely due to the extreme heterogeneity of CRC tumors and rare CSCs in the population.

Genes related to telomerase and telomere recombination (a term with gene list from GSEA) were highly enriched in EPCs_B cells (Figure 3h). The components of telomerase complex *DKC1* and *NOP10*, telomere shelterin complex *TERF1*/*TRF1*, and recombination related genes, such as *POLD2*, *RAD51*, *RAD50*, *FEN1*, *SMC5*, and *SMC6* were highly expressed in EPCs_B cells (Figure 3h). Moreover, EPCs_B cells also expressed *MYC*, a known activator of telomerase, and EPCs_A cells also expressed *MYC* at relatively higher levels, in contrast to CSCs. These data suggest that both telomerase activation and recombination‐based pathway during cell division coincided with the longer telomeres of proliferative EPCs_B cells. Altogether, our data indicates that CSCs of CRC are dormant and exhibit similarities to and differences from cancer EPCs. Dormant state without proliferation presumably grants privilege for CSCs to maintain short telomeres without further shortening.

### 2.7 Plasticity of CSCs and EPCs in CRC

**Figure 4:**
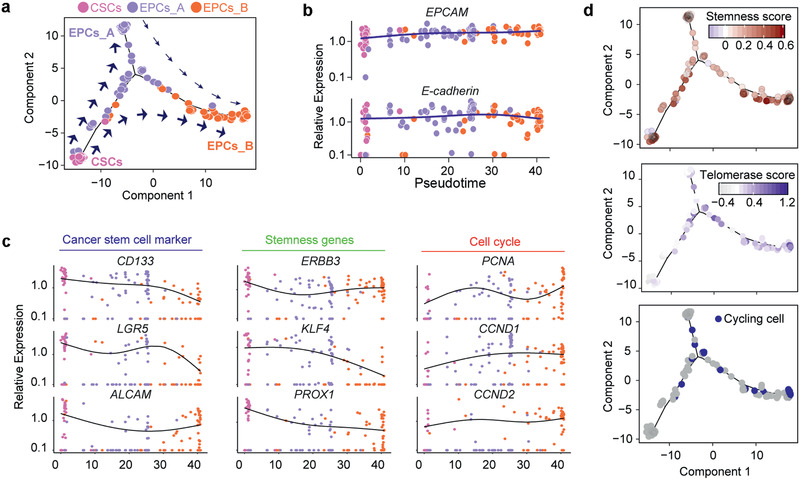
Lineage tracing of CSCs and EPCs. a) Single‐cell trajectories by Monocle analysis showing the development of the “Epithelial lineage” cells. The distance from a cell to the root corresponds to pseudotime. Branched trajectories are plotted as a 2D tree layout. b) Expression of known epithelial markers *EPCAM* and *E‐CADHERIN* with pseudotime. The lines with blue represent the epithelial branch. Each point corresponds to a single cell, and each color represents subpopulation. c) Gene expression levels in single cells ordered along the pseudotime axis for CSC markers, stemness and cell cycle related genes. Cells are color‐coded by subpopulation. The lines indicate local polynomial regression smoothing. d) Distribution of stemness score, cycling cells, and predicted telomerase score with pseudotime exhibit continuous pattern. The color key represents the score levels by a gradient.

To understand how CSCs and non‐CSCs cell types and states are related to each other, we next reconstructed their trajectories by pseudotemporal ordering of single cells using *Monocle*,^[^ ^34^ ^]^ an unsupervised algorithm for inferring branching of lineage assignments and developmental distances using scRNA‐seq data. Remarkably, *Monocle* ordering of CSCs, EPCs_A, and EPCs_B using all detected genes, revealed a trajectory that started from the resting CSC stage and differentiated into the two subtypes of EPCs, indicating gene expression dynamics that recapitulate activation of CSCs (**Figure** **4**a).

Indeed, CSCs and EPCs highly expressed widely reported markers of epithelial lineage cells, such as *EPCAM* and *E‐CADHERIN*/*CDH1* independent of pseudotime path (Figure 4b), and these distinct EPC markers showed continuous high expression during transition of CSCs to EPCs branch. The stemness genes (e.g., *CD133*, *LGR5*, *ALCAM*, *KLF4*, and *PROX1*) were decreased during this transition process along pseudotime path, while the markers of the cell cycle (e.g., *PCNA*, *CCND1*, and *CCND2*) were increasingly expressed (Figure 4c). In addition, consistent with the above data of individual gene, the stemness score decreased from CSCs to EPCs_B cells, while the number of cycling cells increased (Figure 4d). The stemness score was significantly correlated with telomerase activity in EPCs on single cell level (Figure 3f), but not in CSCs. We interpret that the activated EPCs require higher telomerase to support their proliferation and stemness for tumor formation. We then compared cells between CSCs and EPCs, and identified 936 upregulated and 5815 downregulated genes (Figure S4e, Supporting Information). The gene ontology analysis illustrated that up‐regulated genes in CSCs were significantly enriched for several disease‐related terms, such as cell adhesion molecules, T cell receptor signaling pathway, and chemokine signaling pathway (Figure S4f, Supporting Information).

### 2.8 Further Characterization and Validation of CSCs and EPCs in CRC by High Throughput Single‐Cell RNA‐seq

**Figure 5:**
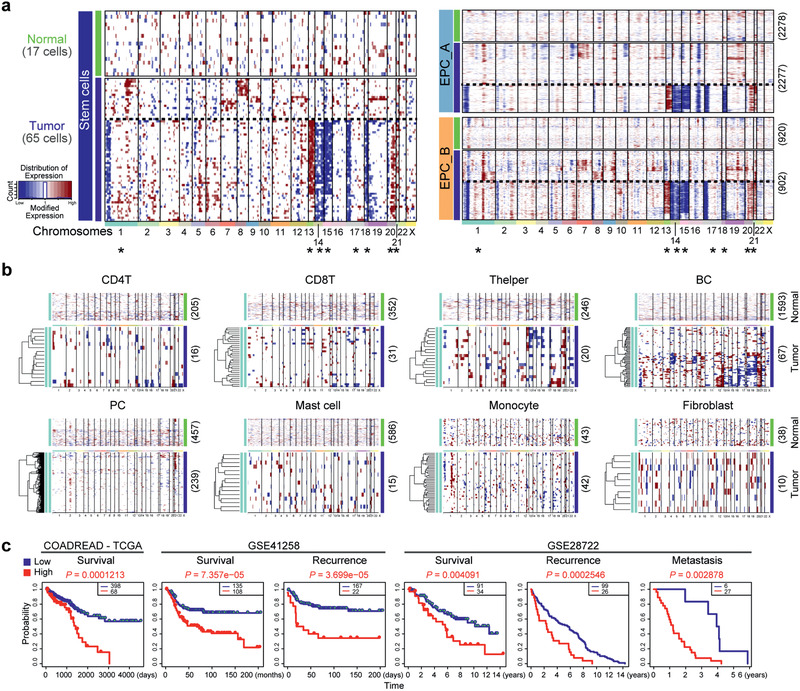
Inferred CNV profiles from 10 × Genomics scRNA‐seq and prediction of prognosis by molecular signature of CSC specific genes. a) Heatmap for chromosomal landscape of inferred large‐scale copy number variations (inferCNVs) distinguishing tumor (malignant) from nontumor cells for individual cells (rows) from the stem cell population and EPCs. Amplifications (red) or deletions (blue) were inferred by averaging expression over 100‐gene stretches on the respective chromosomes (columns). These patterns implicate chromosomal amplification and deletion. * indicate the chromosomes with the same CNVs in CSCs and EPCs. b) Heatmap for chromosomal landscape of inferred large‐scale copy number pattern for individual immune cells (rows) from normal and tumor tissues. c) Survival curve by CSC signature genes. Kaplan–Meier log‐rank tests were performed using default parameters in SurvExpress, an online biomarker validation tool and database (http://bioinformatica.mty.itesm.mx:8080/Biomatec/SurvivaX.jsp). Datasets including The Cancer Genome Atlas (TCGA) (specifically COADREAD‐TCGA (Available for survival), Colon and Rectum adenocarcinoma (Available for survival), GSE41258 (Available for survival and recurrence) and GSE28722 (Available for survival, recurrence and metastasis). Statistical significance was assessed using a log‐rank test.

The aforementioned data from SMART‐seq2 provided in‐depth coverage of transcripts together with telomere length measurement, which limits number of cells analyzed. To validate the features of CSCs and EPCs with a different method, we independently captured a larger number of cells on the 10 × Genomics platform for scRNA‐seq (**Figure** **5** and Figure S5, Supporting Information) from three more CRC patients. In addition to tumors, matched normal tissue from the same patient also was analyzed. We first filtered out the low‐quality cells having transcriptomes with fewer than 200 expressed genes and the genes expressed in less than three cells, such that 3681 cells from tumor tissue and 6735 cells from normal tissue were retained for subsequent analysis (Figure S5a, Supporting Information). The mapping results showed that an average of approximately 7428 unique molecule identifiers (UMIs) from ≈1911 genes were detected per cell (Figure S5a,b, Supporting Information).

By t‐SNE clustering analysis, we identified 11 distinct clusters, including *CD4^+^* T cells (TC) and *CD8^+^* TC, B cell (BC), plasma cell (PC), T helper cells (T helper), fibroblast, mast cells, monocyte, two types of EPCs (EPC_A and EPC_B), and stem cells (SC) (Figure S5c, Supporting Information). Like the SMART‐seq2 data, the specific markers of each cell type were clearly distinguished by the clusters (Figure S5d,e, Supporting Information). We noticed that all 11 clusters identified from the 10 × Genomics data were observed in the three patients, although they varied in their proportions (Figure S5f, Supporting Information). These data indicate that the cell types identified do not represent patient‐specific subpopulations or batch effects.

Remarkably, the proportion of stem cells in tumor tissue increased by seven‐fold more than normal tissue at an average of 1.77% versus 0.25% (Figure S5f, Supporting Information). In addition, the proportion of EPCs in tumor tissue (86%) also nearly doubled comparing to normal tissue (47%) (Figure S5f, Supporting Information). To further mine the molecular features of the stem cells, we analyzed the specific expression of genes derived from the 10 × Genomics data by KEGG analysis for each group. The featured genes were again enriched in WNT, HIPPO, and TGF‐*β* signaling pathway (Figure S5g, Supporting Information). These pathways were also associated with the genes enriched in CSCs independently revealed by our SMART‐seq2 data. Consistently, stem cell groups characterized by 10 × Genomics also exhibited higher stemness score than other cell types, while the terminally differentiated plasma cell (PC) received the lowest stemness score (Figure S5h, Supporting Information). The markers for stem cell group identified from 10 × Genomics were shared in large number with those of SMART‐seq2 and these shared genes enriched in the same functional terms related to stemness (Figure S5i, Supporting Information). These data highlight that the data from our 10 × Genomics and SMART‐seq2 are reasonably consistent.

Comparison of the two different stem cells, tumor stem cells and normal stem cells, showed that the pathways of mTORC1, MYC signaling and oxidative phosphorylation, were positively enriched in tumor stem cells (Figure S5j, left panel, Supporting Information). mTORC1 activation is essential for the cells that require adequate energy resources, nutrient availability, oxygen abundance, and proper growth factors for mRNA translation to begin.^[^ ^35^ ^]^ In addition, cell function of the upregulated genes in tumor stem cells was more enriched with activation in the immune responses (Figure S5j, right panel, Supporting Information). Accordingly, cell‐cycle status analysis indicated that stem cells from tumor tissue are noncycling cells (Figure S5k, Supporting Information), consistent with their quiescent state. While more proliferating cells were found in EPC_B cells, and MKI67 labeled proliferating cells also were mainly enriched in EPC_B cells (Figure S5k, Supporting Information). We observed some residual MKI67+ cells in EPC_A cells, and we infer that these rare MKI67+ cells can be just transited from EPC_B with a low proliferation activity. In addition, compared to the normal stem cells, CSCs specifically expressed genes including *SMOC2*, *BAMBI* and *NOTUM* (Figure S5l, Supporting Information), consistent with our SMART‐seq2 data. Hence, single‐cell analysis of a larger number of cells reveals that CSCs are distinct from normal stem cells.

Genomic mutation or stability, shown as copy‐number variations (CNVs), including amplifications, deletions, and whole‐chromosome gains or losses, is a hallmark of cancer.^[^ ^36^ ^]^ To distinguish CSCs from normal tissue stem cells, we attempted to obtain patterns of large‐scale CNVs for each stem cell by averaging relative expression levels over large genomic regions along with conventional single‐cell transcriptome profiling.^[^ ^1^, ^37^ ^]^ The normal sample with 17 single cells served as controls. Approximately 67% (44 of 65) of the stem cells from tumor exhibited distinct chromosomal gene expression pattern (higher CNV levels) and normal stem cells showed no apparent CNVs (Figure 5a). To define malignant cells of two EPC cell types, we calculated the CNV scores in each cell across the genome following the same assay, and found that over 40% (41% of EPC_A and 55% of EPC_B) of EPCs from tumor tissues were featured with high CNVs while the EPCs from normal control tissues apparently without CNVs. Interestingly, it showed that the stem cells in tumors acquired mutations by increased CNVs notably on chromosome 1, 13, 14, 15, 17, 18, 20, and 21, distinct from stem cells in normal tissue. Coincidently, about 40% of EPC_A and EPC_B cells (less than 50% in EPA_A and more than 50% in EPC_B) exhibited the same patterns of CNVs on the same chromosomes as that in the CSCs (Figure 5a). These results suggested that the stem cells and EPCs beard CNVs in cancer fit the real CSCs and malignant cancer EPCs, and that CSC and cancer CSC share the same originality. On the other hand, the stem cells and EPCs without CNVs are their healthy counterpart in normal tissues or tumor tissues. Gain of chromosome 13 and loss of chromosome 14 and 15, the most common genetic alterations in human colon and rectal cancer,^[^ ^30^ ^]^ were consistently inferred from our 10 × Genomics results (Figure 5a). We also inferred CNVs for several other cell noncancer populations both in normal and tumor tissues. These cell types display “normal” (healthy) CNV patterns with only some minor noise signals, which are the baseline technical noise, with apparent exception B cells (Figure 5b). Nevertheless, these possible CNVs differed from those observed in CSCs and cancer EPCs.

Taken together, the similar outcomes obtained from different RNA‐seq methods further validate the common features of CSCs and EPCs. Moreover, based on the connection in stemness, shared marker genes and signaling pathways but distinctions in the mutation and specific genes for CSCs, the CSCs of CRC may originate from normal stem cells at the tumor initiation state.

### 2.9 Association of the Signature of CSCs and EPCs with Prognosis of CRCs

We explored the possibility that the cell‐type molecular signatures, particularly those shared by CSCs and EPCs, correlate with the clinical outcome of CRCs. First, we looked at whether the top highly expressed genes in CSCs defined above were associated with clinical outcome, using the public datasets from The Cancer Genome Atlas (TCGA), specifically COADREAD‐TCGA, Colon, and Rectum adenocarcinoma, GSE41258 and GSE28722, and the survival analysis software *SurvExpress*.^[^ ^38^ ^]^ Since CSCs feature the traditional stem cell properties with therapeutic resistance, we attempted to test whether signature genes of CSCs in comparison with normal stem cells can predict poor outcome of CRC patients. Indeed, patients with high expression levels of CSC specific genes showed low survival in the all query datasets, with significant correlation with recurrence (GSE41258 and GSE28722) and metastasis of CRCs (GSE28722) (Figure 5c).

We also compared expression profiles of malignant and less malignant tumor tissue from CRCs using bulk expression profiles from TCGA data sets and categorized them into 11 distinct microenvironment signatures based on their inferred cell type composition. Cancer samples with a high abundance of cancer EPC signatures (CSCs, EPCs_A and EPCs_B) were malignant tumor samples, whereas cancer samples with fewer signatures were more frequently included in less malignant samples (Figure S6a, Supporting Information). These results also suggest that the signature of CSC and EPC abundance is linked to preferential expression in the poorly differentiated tumors; therefore these signatures may be used for prognosis. These additional data analyses further validate our characterization of CSCs and EPCs at the single‐cell resolution above.

Moreover, CRC patients with a high CSC signature represented by the CSC specific genes (*NOTUM*, *SMOC2*, *BAMBI*, *PHLDA1*, *TNFRSF19*, *PROX1*, *IFI27*, and *ERBB3*), exhibited significantly worse survival (Figure S6b left, Supporting Information). Six up‐regulated genes (*EPCAM*, *CDH1*, *KRT18*, *CLDN4*, *CXADR*, and *SLC12A2*) selected based on our functional analysis above and shared by both CSCs and EPCs in SMART‐seq2 and 10 × Genomics data, predicted the poorest survival rate of CRC patients (*P* = 1.686 × 10^−5^, Figure S6b right, Supporting Information). We also performed a similar analysis using the top 20 highly expressed signature genes in each subpopulation, and found only a weak correlation in T cells, macrophages and dendritic cells and no correlation in the rest of the cell populations (Figure S6c, Supporting Information). These data show that the specific signature genes of CSCs predict poor clinical outcome of CRC patients and could potentially become clinically useful prognostic biomarkers.

### 2.10 Experimental Validation and Significance of the Signature Genes in CSCs and EPCs

We validated the function of these specific genes in CSCs and EPCs from human CRC primary tumors. Using the seven signature genes shared by both CSCs and EPCs, we by reverse transcription qRT‐qPCR compared their expression levels in three patients with recurrence (poor prognosis) and three patients without recurrence (well prognosis) at three years after surgery. We observed elevated expression levels of these genes in the poor prognosis group despite the few samples available (*P* = 0.0237; Figure S6d,e, Supporting Information). Of these key signature genes, *EPCAM* and *CD133* reportedly are expressed at high levels in human CRCs and associated with prognosis.^[^ ^39^ ^]^ The other four enriched genes *KRT18, SLC12A2, CXADR*, and *CLDN4* also were expressed at higher levels by immunofluorescence in patients with poor prognosis compared to patients with well prognosis (Figure S6e, Supporting Information).

*KRT18* (cytokeratin 18, also named *CK18*) encodes for the type I intermediate filament chain keratin 18, and regulation of *KRT18* by WNT is involved in AKT activation.^[^ ^40^ ^]^ In addition, *KRT18* plays an important role in chemo‐sensitivity in lung cancer.^[^ ^41^ ^]^ *KRT18* was highly expressed in the tumor site as assessed by both RT‐qPCR and immunofluorescence (Figure S6d,e, Supporting Information). KRT18 could be an important tumor marker for clinical diagnosis of CRCs. SLC12A2 is a blocker of Na^+^/K^+^/2Cl^−^ cotransporter (NKCC), and over‐expression of *SLC12A2* promotes cell proliferation and correlates with poor differentiation and metastasis of tumor cells.^[^ ^42^ ^]^ Higher levels of *SLC12A2* were found in poor prognosis compared with well prognosis samples, which displayed relatively reduced expression signals (Figure S6e, Supporting Information). CXADR (coxsackie virus and adenovirus receptor) is localized to epithelial tight junctions in vivo where it may regulate epithelial permeability and tissue homeostasis.^[^ ^43^ ^]^ *CXADR* was also highly expressed in the poor prognosis samples (Figure S6e, Supporting Information), and may be closely involved in EPC functional maintenance. *CLDN4* was highly expressed in both CSCs and EPCs, and correlated with prognosis (Figure S6e, Supporting Information). The claudin (CLDN) family of transmembrane proteins plays a critical role in the maintenance of epithelial and endothelial tight junctions and promotes self‐renewal of human CRC stem‐like cells.^[^ ^44^ ^]^

**Figure 6:**
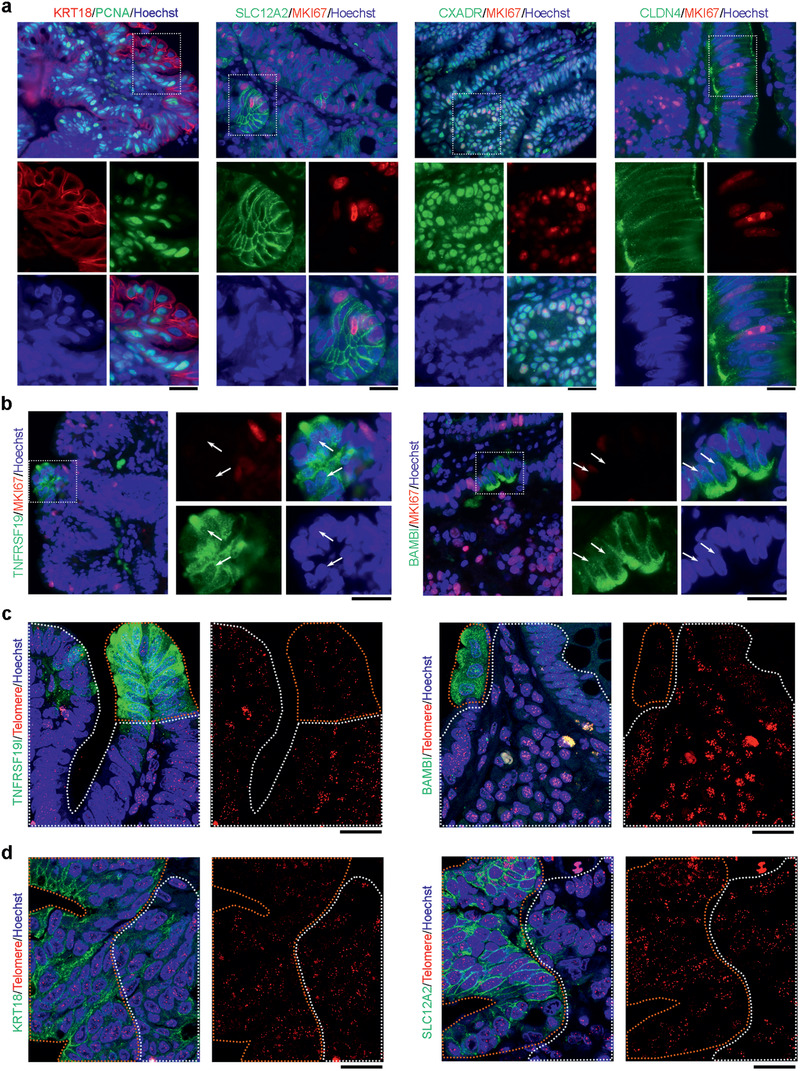
Validation of enriched genes shared by CSCs and EPCs or specific for CSCs by immunofluorescence and telomere FISH of CRCs. a) Proliferation markers PCNA or MKI67 and shared markers KRT18, SLC12A2, CXADR, and CLDN4. b) Proliferation marker MKI67 costained with CSC enriched genes, TNFRSF19 and BAMBI. White arrows indicate marker positive but MKI67 negative cells. c) CSC specific markers TNFRSF19 or BAMBI (green) and telomere FISH (red). Areas within dashed lines in orange indicate marker positive cells; areas within dashed lines in white indicate marker negative cells. d) CSC and EPC shared markers SLC12A2 or KRT18 and telomere FISH (red). Areas within dashed lines in orange indicate marker positive cells; areas within dashed lines in white indicate marker negative cells. Scale bar = 20 µm.

To further validate the features of cells identified by shared and CSC‐specific gene expression revealed by our simultaneous analysis of the transcriptome and telomere length, we visualized these markers and telomere length in tumor sections by immunofluorescence microscopy with telomere fluorescence in situ hybridization (IF‐FISH). The markers KRT18, SLC12A2, CXADR, and CLDN4 shared by CSCs and EPCs showed a noticeable wide distribution pattern with high proliferation cells marked by PCNA or MKI67 expression in CRC tumors (**Figure** **6**a). In contrast, BAMBI and TNFRSF19 positive cells from primary tumor sections did not express or only minimally expressed MKI67 (Figure 6b). Cells positive for BAMBI and TNFRSF19 were rare, about 2.5% and 1.6%, respectively (after counting more than 5000 cells from 7 patients). Of these positive cells, more than 70% BAMBI positive cells and 90% TNFRSF19 positive cells were negative for MKI67 in these tumors (Figure 6b). These immunofluorescence in situ data were consistent with our single‐cell analysis results that proliferation is low in CSCs. IF‐FISH of tumors using CSC feature genes (e.g., BAMBI and TNFRSF19) showed that telomeres were notably shorter in a subset of the marker positive cells than in negative cells (Figure 6c), validating short telomeres in CSCs delineated by our simultaneous single‐cell analysis of telomeres and transcriptome. However, cells positive for markers, such as KRT18 and SLC12A2, shared by CSCs and EPCs exhibited heterogeneous telomere lengths (Figure 6d). This was not unexpected since EPCs would contain relatively longer telomeres and CSCs shorter telomeres, based on the combined single‐cell analysis results.

**Figure 7:**
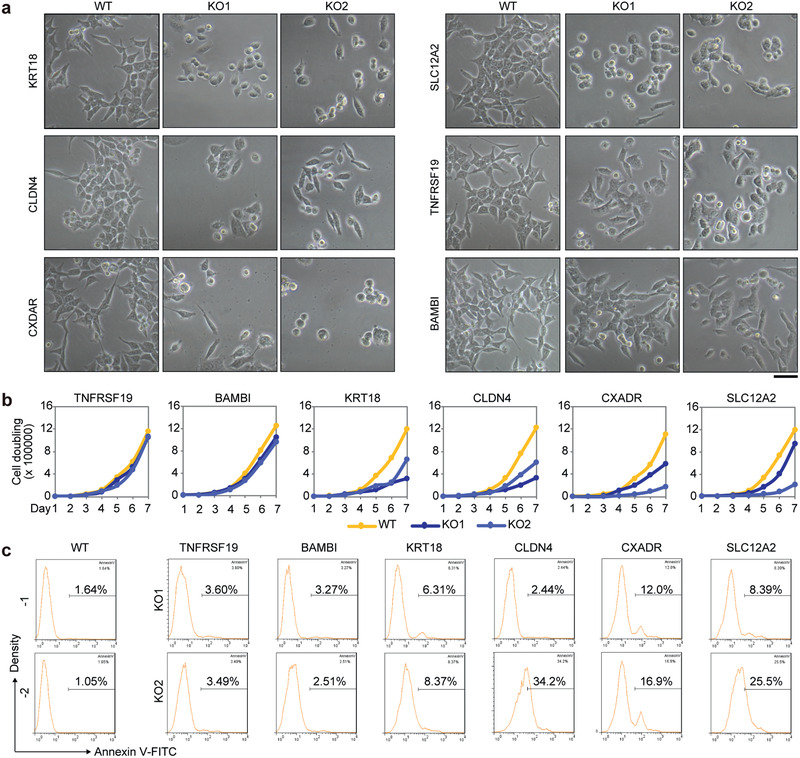
Functional analysis of specific genes in CSCs or shared by both CSCs and EPCs by CRISPR/Cas9. a) Morphology of cells with knock‐out of selected key genes. Two sgRNAs were used for construct knockout (KO) cell lines on human CRC HCT116 by Lipo2000 system, and stable KO clones were selected after sorted the GFP positive cells by FACS. Scale bar = 50 µm. b) Growth curves of cells with key gene knockout. Approximately 1 × 10^4^ HCT116 cells (at passage 5) were seeded on Day 1. Cells were counted every day. *n* = 3 repeats. c) Percentage of apoptotic cells in each group (at passage 5) was detected by flow cytometry using Annexin V‐FITC.

Furthermore, we tested the potential cellular functions of a set of selective marker genes, including the four new prognosis‐valuable genes (*KRT18*, *CLDN4*, *CXADR*, and *SLC12A2*) shared by both EPCs and CSCs, and two specific genes *TNFRSF19* and *BAMBI* enriched in CSCs, using HCT116 cell line by CRISPR/Cas9 dual sgRNA‐mediated gene deletion (Figure S7a, Supporting Information). HCT116 cell line exhibits CSC‐like properties and has been used as a model to study the functions of human CRC cell stemness.^[^ ^45^ ^]^ In addition to Sanger sequencing validation of the gene knock‐out cell lines (Figure S7b, Supporting Information), the loss of corresponding protein was also validated by Western blot analysis (Figure S7c, Supporting Information). Deletion of *KRT18, CXADR, CLDN4*, or *SLC12A2* resulted in reduction of cell size and the cells showed round‐shaped cell in morphology, perhaps suggestive of the cells undergoing cell death, but deletion of *TNFRSF19* or *BAMBI* showed less effect on cell morphology (**Figure** **7**a). Functional disruption of *KRT18, CXADR, SLC12A2*, or *CLDN4* significantly reduced cell proliferation, but interestingly *TNFRSF19* or *BAMBI* disruption did not (Figure 7b). Further analysis of cell apoptosis by Annexin V staining indicated that deletion of *KRT18, CXADR, SLC12A2*, or *CLDN4* resulted in increased apoptosis and necrosis (Figure 7c). These data provide additional functional evidence to validate the signature of dormant CSCs revealed by our comprehensive analysis of telomeres and transcriptome in the same cell, also supporting the notion that it is the dormant CSCs that resist chemo‐therapy and may initiate recurrence when being activated to enter proliferation, via transforming to EPCs.

## 3 Discussion

Our data reveals that rare CSCs in CRCs exist in a dormant state and possess high stemness and high WNT, TGF‐*β* and YAP/HIPPO signaling and are able to maintain short telomeres without cell proliferation. However, these rare dormant CSCs and the more cancerous EPCs share the common cell surface markers that are conventionally used to identify CSCs, and these data may explain the various frequencies reported for CSCs.^[^ ^3^, ^4^, ^5^ ^]^ A portion of EPCs can be tracked from dormant CSCs by lineage tracing and mutation origins, acquire telomerase activity and elongate telomeres and may represent proliferative TICs,^[^ ^3^, ^8^ ^]^ resulting in high self‐renewal activity or transit‐amplifying cells (Figure S8, Supporting Information).

On the assumption that rare CSCs potentially exist in primary tumors, we initially enriched CSCs by FACS using commonly known cell surface markers CD133, CD44, and/or LGR5, or tumorsphere formation. Yet, CD133, CD44 and/or LGR5 positive or negative cells are detected in both CSCs and EPCs, suggesting that the common cell surface markers alone could not indicate nor distinguish CSCs from EPCs. CD44 or its isoform has long been thought to be a marker CSC marker,^[^ ^4^, ^46^ ^]^ however, controversy remains especially as to its correlation with clinical outcome.^[^ ^39^, ^47^ ^]^ In addition, single‐cell cloning of colon CSCs revealed a multilineage differentiation capacity in tumorigenesis using CD44^−/−^ mice model.^[^ ^29^ ^]^ From the 10 × Genomics data with all cells, the population of EPC_A plus EPC_B are ≈2‐fold in tumor tissues than in the matched normal colon tissues, and about 40% EPCs in tumors were likely malignant cancer EPCs. Meanwhile only 1.77% cells in tumor tissues are of the quiescent stem cells and 70% of these stem cells are CSC population. These observations also suggest that identification of rare CSCs is limited by FACS using existing surface markers, which is insufficient to recover the wide heterogeneity of CSCs. Recently, single‐cell analysis of the transcriptome and epigenome also showed that *KRT8, KRT18, CLDN4, KRT19, KRT20*, etc. are highly expressed in CRCs,^[^ ^12a^ ^]^ consistent with our results that these genes were highly expressed in cancer EPCs. Our integrated single‐cell analysis of telomere length and transcriptome profile in the same cell is essential to the discovery of CSCs and their features, and to clarify their nexus to cancer EPCs (Figure S8, Supporting Information).

Additionally, high throughput scRNA‐seq on 10 × Genomics reveals that stem cells from tumor and normal can be clustered together, but the stem cells from a tumor accumulate CNVs and acquire activation signals to the immune response than do normal stem cells. Most recently, a combination of single cell analysis by SMART‐seq2 and 10 × Genomics scRNA‐seq validated tumor‐infiltrating immune cells and their cellular interactions for regulating tumor.^[^ ^48^ ^]^ Another exciting study by single cell analysis of mouse and human CRCs also uncovered tumor initiating stem cells that display high YAP, regulated by signaling from a mesenchymal niche.^[^ ^49^ ^]^ These findings further demonstrate the power of single cell analysis in the discovery of new cell types and their interactions and conversions.

CSCs may originate from normal tissue stem cells following mutations.^[^ ^5^, ^8^ ^]^ Here a systematic investigation by SMART‐seq2 and validation by 10 × Genomics scRNA‐seq consistently demonstrate that stem cells in CRC appear to have mutated and nonmutated populations, and the mutated populations share the same mutation patterns as a part of EPCs in cancer. We suggest that these mutated stem cells are CSCs and they are the progenitors of cancer EPCs, the malignant EPCs in cancer tissues (Figure 5). This conclusion is supported by the lineage tracing analysis (Figure 4), whereas other EPCs without evident mutations look more like normal EPCs. CSCs and EPCs exhibit high YAP and WNT signaling activity, driven by mutation of related genes, such as *APC* and *KRAS*, which has been identified to be critical for CRC tumorigenesis.^[^ ^30^, ^50^ ^]^ CSCs may also develop through acquisition of stemness from differentiated cells by reprogramming during tumor progression.^[^ ^51^ ^]^ CSCs have short telomeres but without further shortening, and this may be explained by their dormancy without cell proliferation, such that short telomeres do not undergo further shortening without telomerase. Recently, we show that telomeres are short in CSC‐like cells and the length is maintained by PML based mechanisms.^[^ ^52^ ^]^ It remains to be clarified how dormant CSCs acquire telomerase and become proliferative CSC‐like cells, EPCs. Although both dormant CSCs and active EPCs share the signaling pathways of YAP, WNT and TGF‐*β*, TGF‐*β* signaling is more active in CSCs than in EPCs. Interestingly, YAP and WNT can activate telomerase,^[^ ^53^ ^]^ while TGF‐*β* inhibits *TERT* expression and telomerase activity.^[^ ^54^ ^]^ Higher TGF‐*β* signaling in CSCs is consistent with their minimal telomerase activity and quiescent state. Nonetheless, dormant CSCs themselves presumably do not directly initiate tumorigenesis unless certain signals, stimulus or niche that remain to be determined are provided to waken the cells.

The cellular plasticity has been reported in healthy intestine^[^ ^28^, ^55^ ^]^ and the stem cell plasticity is tightly linked to changes in WNT levels,^[^ ^56^ ^]^ while cancer cells also can dynamically shift between a differentiated and a stem‐like state.^[^ ^1c^ ^]^ Even the LGR5 negative cells possess the intrinsic capacity for rebuilding the epithelial hierarchy organization.^[^ ^57^ ^]^ CSCs are often embedded in and thus confused with EPCs.^[^ ^57a^ ^]^ Here, we found that quiescent CSCs also exhibit high plasticity and can generate EPCs. Higher stemness, low cell proliferation, low telomerase activity and maintenance of short telomeres provide unique features of dormant CSCs that can be used to discern them from proliferative EPCs, and these features of plasticity may allow CSCs to pioneer resistance to therapy as well as recurrence. The dormancy of CSCs or CSC‐like cells also have been found in other cancers, including breast melanoma, and glioblastoma.^[^ ^58^ ^]^ We show that both dormant CSCs and proliferative EPCs highly express LGR5 and here link the dormant CSCs in CRC to proliferative EPCs as having been designated as TICs or tumor‐initiating stem cells.^[^ ^59^ ^]^

High telomerase activity and sufficient telomeres are essential for self‐renewal of tumorigenesis.^[^ ^13a,b^ ^]^ Therefore, there is a persistent effort to develop therapeutics that is telomerase‐specific but gentle to nonmalignant tissues.^[^ ^14c^ ^]^ Dormant CSCs with low telomerase activity and short telomeres can maintain the stem cell pool and supply the proliferative EPCs, yet are resistant to chemo‐ or radiation‐theray. Interestingly, TICs from a panel of prostate cancer cell lines or glioblastoma TICs have significant telomerase activity, which can be effectively inhibited by telomerase inhibition.^[^ ^60^ ^]^ It seems that EPCs exhibit features of TICs. Targeting telomerase using specific inhibitors could prove effective in demolishing EPCs or TICs with high telomerase activity, whereas quiescent CSCs with minimal telomerase activity may evade the therapy. Regardless, identification of CSCs at different states and their lineage tracing should facilitate targeted therapies to the tumor origin and progression.

## 4 Experimental Section

##### Human Primary Colorectal Tumors

Human primary colorectal tumors for scT&R‐seq were obtained from Tianjin Medical University General Hospital. This study was approved by the Ethical Committee at Tianjin Medical University General Hospital, and informed consent was obtained prior to investigation. Tumor isolation was carried out as described previously.^[^ ^3a^ ^]^ Tumor tissues were isolated from fresh CRCs within 2 h after collection and were minced into small chunks (≈2 mm^3^), and then intensively washed six times in cold PBS containing penicillin (500 U mL^−1^), streptomycin (500 µg mL^−1^), and amphotericin B (1.25 µg mL^−1^; E485, Amresco). Four male and four female patients were used, and the information is provided in Table S1 (Supporting Information). For 10 × Genomics analysis, primary colorectal carcinomas and their corresponding control tissues as well as clinical information were provided by the Tissue Bank of Yale‐New Haven Hospital (Table S10, Supporting Information). All patients were deidentified and the standard operation procedures were approved by the Management Committee at Department of Pathology, Yale University School of Medicine (YSOP#100).

##### Tumor Dissociation

Tumor samples collected from primary surgical specimens were mechanically and enzymatically disaggregated into single‐cell suspensions, following previously published protocols.^[^ ^10^ ^]^ Briefly, a single‐cell suspension of washed fresh biopsy sample was prepared by mincing the tissues with scissors into small fragments (0.2–0.5 mm^3^), followed by incubation in advanced DMEM/F12 (12634‐010, Life Technologies) with 1% penicillin/streptomycin (15140‐122, Invitrogen), 1.5 mg mL^−1^ (75 U mL^−1^) collagenase type IV (17104019, Life technologies), 20 µg mL^−1^ Hyaluronidase (H‐6254, Sigma), 10 × 10^−6^ m Y‐27632 (281642A, Santa Cruz), and DNase I (D5025‐15KU, Sigma) for 30 min at 37 °C to obtain enzymatic disaggregation, which was followed by filtration through a 40 µm cell strainer (352340, BD Biosciences). Red blood cells were removed by 1 × red blood cell (RBC) lysis buffer (00‐4300‐54, eBioscience) for 10 min, and dissociated cells were pelleted and re‐suspended in 0.1% bovine serum albumin (BSA, A3059‐10G, Sigma) with 10 × 10^−6^ m Y‐27632.

##### Cell Line

The human colorectal cancer cell line HCT116 (RRID: CVCL_0291) cells were cultured at 37 °C in 5% CO_2_ in RPMI 1640 (11875085, Life technologies) plus 10% FBS and penicillin (100 U mL^−1^) and streptomycin (100 µg mL^−1^), as previously described.^[^ ^61^ ^]^

##### Fluorescence‐Activated Cell Sorting

To minimize loss of cell viability, the following experiments on fresh cell suspensions were performed, prepared shortly before flow cytometry. The primary cells were stained with anti‐LGR5‐APC antibody (562903, BD Biosciences), anti‐CD133‐APC (130‐098‐129, Miltenyi Biotec), or anti‐CD44‐FITC (H20441‐02H, Sungene Biotech) for 20 min on ice in 0.1% BSA/PBS, at a concentration of 5 × 10^5^ cells/100 µL, then washed with 3.5 mL 0.1% BSA/PBS and resuspended in 1 mL 0.1% BSA/PBS containing 50 *μ*L 7‐aminoactinomycin D (7‐AAD, 559925, BD Biosciences) and 10 × 10^−6^ m Y‐27632 for flow cytometry analyses. Fluorescence‐activated cell sorting (FACS) was achieved by AriaII cell sorter (BD Biosciences) equipped with 488 nm (FITC) and 633 nm (APC) lasers. Side‐scatter area (SSC‐A) versus forward‐scatter area (FSC‐A) and FSC‐A versus forward‐scatter width profiles were used to discard doublets and capture singlets. Dead cells were eliminated by excluding 7‐AAD^+^ (PerCP channel) cells.

##### Tumorsphere Formation

Sphere medium for human colorectal cancer was prepared as previously described.^[^ ^8b^ ^]^ Briefly, after blood cells were removed by lysis buffer, the cell pellet was suspended with sphere culture medium and dispensed into a low attachment well plate (3471, Corning) for tumorsphere formation. Tumor cells were placed in serum‐free DMEM/F12 (12634‐010, Invitrogen) supplemented with human recombinant EGF (AF‐100‐15, PeproTech) and basic FGF (PHG0026, Life technologies) with 10 × 10^−6^ m Y‐27632, growth factors were added every other day and medium was changed every 7 days. Tumorspheres were then disaggregated to single cells after 14 days for single‐cell RNA‐seq.

##### Single‐Cell Isolation and Separation of Genome and Transcriptome in the Same Cell

Single cells were picked up in 1 *μ*L 0.1% BSA/PBS using a micropipette with an epT.I.P.S. Pipette tips (0030000838, Eppendorf) under a dissecting microscope and transferred to the bottom of a 200 µL PCR tube (8‐strip, nuclease‐free, thin‐walled PCR tubes with caps, PCR‐0208‐C, Axygen) with 4 *μ*L cell lysis buffer which contained 3.45 *μ*L of Buffer RLT plus (1053393, Qiagen), 0.5 *μ*L Biotin‐oligo‐dT reverse transcription (RT) primer (5ʹ‐biotin‐TEG‐AAGCAGTGGTATCAACGCAGAGTACT_30_VN‐3ʹ) and 0.05 *μ*L of Recombinant RNase inhibitor (2313A, Clontech). Consortium RNA spike‐in Mix (ERCC, 4456740, Life Technologies) was added to the lysis reaction and processed in parallel to cellular mRNA. Separation of gDNA and mRNA was performed as described previously.^[^ ^62^ ^]^ Briefly, samples were incubated at 72 °C for 3 min, and Biotin‐oligo‐dT primer was hybridized to poly (A)^+^ tail mRNA. Dynabeads (65001, Life technologies) in 4 *μ*l were added to capture mRNA, and collected by a magnet for scRNA‐seq, and gDNA supernatant was transferred to a fresh PCR tube for telomere length measurement.

##### Single‐Cell RNA Sequencing: Reverse Transcription

Single‐cell cDNA was synthesized from the tubes containing mRNA according to SMART‐seq2 protocol,^[^ ^63^ ^]^ with minor modifications: Dynabeads containing single‐cell mRNA were suspended in every PCR tube with 10 *μ*L of the following RT Mix: 0.25 *μ*L 100 × 10^−6^ m template‐switching oligonucleotides (TSO) primer (5ʹ‐AAGCAGTGGTATCAACGCAGAGTACATrGrG+G‐3ʹ), 1 *μ*L 10 × 10^−3^ m dNTP (11969064001, Roche), 0.06 *μ*L 1 m MgCl_2_ (AM9530G, Ambion), 2 *μ*L 5M betaine (77 507, Affymetrix), 0.5 *μ*L 100 × 10^−3^ m DTT, 2 *μ*L 5 × Superscript II First‐Strand Buffer, 0.5 *μ*L 200 U *μ*L^−1^ SuperScript II reverse transcriptase (18064014, Invitrogen), 0.25 *μ*L 40 U *μ*L^−1^ RNAse inhibitor (2313A, Clontech) and 3.44 *μ*L nuclease‐free water (W4502‐1L, Sigma‐Aldrich). Every 8‐strip PCR tube was mixed with the beads by vortex. Reverse transcription was carried out by incubating the tubes at 42 °C for 90 min, followed by 10 cycles of 50 °C for 2 min and 42 °C for 2 min. Finally, the reverse transcriptase was inactivated at 70 °C for 15 min.

##### Single‐Cell RNA Sequencing: PCR Preamplification and cDNA Purification

Fifteen microliters of the following PCR preamplification mix was added in each well: 0.5 *μ*L 10 ×10^−3^ m PCR pre‐amplification primer, 12.5 *μ*L 2 × KAPA HiFi Mix (KK2601, KAPA Biosystems) and 2 *μ*L nuclease‐free water, with a final PCR reaction volume of 25 *μ*L. The preamplification program used was as follows: 98 °C for 3 min, 22 cycles of 98 °C for 15 s, 67 °C for 20 s and 72 °C for 6 min, with a final extension at 72 °C for 5 min. PCR products were purified by mixing with 25 *μ*L (1 ×) of Agencourt AMPureXP SPRI beads (A63881, Beckman‐Coulter), followed by incubation for 8 min. The tubes were then placed onto a magnet (A29164, Agencourt) for 8 min and the supernatant removed by careful pipetting. SPRI beads (1×) were washed twice with 200 *μ*L of freshly prepared 80% ethanol. Upon removing all residual ethanol traces, SPRI beads were left to dry at room temperature for 10 min. The beads were then resuspended in 25 *μ*L of elution buffer (19086, Qiagen) and incubated at room temperature for 5 min. The plate was placed on the magnet for 8 min and the supernatant containing the amplified cDNA was transferred to a new 8‐strip PCR tube. The concentration of amplified cDNA was measured with Qubit 1.0 Fluorometer (Invitrogen) using the Quant‐iT Qubit dsDNA BR Assay Kit (Q32853, Invitrogen). The cDNA size distribution was checked by a High‐Sensitivity DNA chip (Agilent Bioanalyzer) or gel electrophoresis, and only cDNA sharply peaking around 2 kb was used for library preparation.

##### Single‐Cell RNA Sequencing: Library Construction

Libraries were prepared by using TruePrep DNA Library Prep Kit V2 for Illumina (TD503‐02, Vazyme Biotech) according to the product manual, allowing 96 single cell libraries to be simultaneously generated in 12 rows with 8‐strip PCR tubes. The tagmentation reaction consisted of 1 ng purified cDNA with 4 *μ*L of TruePrep Tagment Buffer L and 5 *μ*L of TruePrep Tagment Enzyme, added with purified water to total 20 *μ*L, and mixed well by pipetting 20 times. The reaction was incubated at 55 °C for 12 min. A volume of 5 *μ*L of Terminate Solution was added and the solution was mixed prior to incubation at room temperature for another 5 min. The libraries were amplified by adding 1 *μ*L of TruePrep Amplify Enzyme, 10 *μ*L TruePrep Amplify Buffer, 5 *μ*L of N5 adapter (TD202, Vazyme Biotech), and 5 *μ*L of N7 adapter (TD202, Vazyme Biotech). The PCR was then carried out at an initial incubation at 72 °C for 3 min, 98 °C for 30 s, followed by 14 cycles of 98 °C for 15 s, 60 °C for 30 s and 72 °C for 3 min, and a final extension at 72 °C for 5 min.

Following PCR amplification, each library was purified by Agencourt AMPureXP SPRI beads to generate an Illumina‐compatible sequencing library according to the product manual with final size distribution of 300–700 bp. Briefly, PCR products were purified by mixing them with 30 *μ*L (0.6 ×) of SPRI beads, followed by a 5 min incubation period at room temperature. The plate was then placed onto a magnet for 6 min prior to transferring the supernatant to a new PCR tube. Another 7.5 *μ*L (0.15×) of SPRI beads was added to the new PCR tube, followed by a 5 min incubation at room temperature. The plate was placed onto a magnet for 5 min prior to removing the supernatant. SPRI beads were washed twice with 100 *μ*L of freshly prepared 80% ethanol, with care being taken to avoid loss of beads during the washes. Upon removing all residual ethanol traces, SPRI beads were left to dry at room temperature for 10 min. The beads were then resuspended in 25 *μ*L of nuclease‐free water (W4502‐1L, Sigma‐Aldrich) and incubated at room temperature for 5 min. The plate was placed on the magnet for 5 min prior to transfer of the supernatant containing the selected size of library to a new tube.

##### Single‐Cell RNA Sequencing: Illumina Sequencing

Libraries were pooled after quantification by the KAPA HyperPlus Library Preparation Kit (KK8514, KAPA Biosystems). Multiplexed single‐cell libraries were pooled and sequenced with a 125 bp paired‐end sequencing strategy on a HiSeq 2500 platform (Illumina).

##### Single‐Cell Telomere Length Measurement

The gDNA was purified by 8 *μ*L Agencourt AMPureXP SPRI beads. After wash with 80% (vol/vol) ethanol, gDNA beads were stored at −80 °C. Telomere length of each cell was measured using single‐cell telomere length measurement by quantitative PCR assay (SCT‐pqPCR) as previously described,^[^ ^16^ ^]^ with slight modifications. A multiplex preamplification (pre‐PCR) step that can simultaneously amplify telomere repeats (T) and *Alu* reference gene (R) was employed with the telomere primers (forward primer: CGGTTTGTTTGGGTTTGGGTTTGGGTTTGGGTTTGGGTT, reverse primer: GGCTTGCCTTACCCTTACCCTTACCCTTACCCTTACCCT), and *Alu* primers (forward primer: GACCATCCCGGCTAAAACG, reverse primer: CGGGTTCACGCCATTCTC). The reactions were set up by aliquotting 25 *μ*L of a master mix with single‐cell gDNA beads. Each reaction was set up with 2.5 *μ*L 10 × iTaq buffer, 1.5 × 10^−3^ m MgCl_2_, 0.625 U iTaq DNA polymerase (170‐8870, Bio‐Rad), 0.5 *μ*L 10 × 10^−3^ m dNTP mix (170‐8874, Bio‐Rad), 1 *μ*L each of telomere forward and reverse primer (10 × 10^−6^ m), and 1 *μ*L each of *Alu* forward and reverse primer (10 × 10^−6^ m). Thermal cycler reaction conditions were set at 95 °C for 3 min followed by 18 cycles of 95 °C for 30 s, 60 °C annealing for 30 s and extension at 72 °C for 30 s. The PCR products were purified using 1.8 × Agencourt AMPureXP SPRI beads and eluted in 64 *μ*L double distilled water. iQ SYBR Green‐based real‐time PCR (170‐8882, Bio‐Rad) was performed using the same primers and reaction conditions of 95 °C for 10 min followed by 30 cycles of data collection at 95 °C for 15 s, 60 °C annealing for 30 s and extension at 72 °C for 30 s along with 80 cycles of melting curve from 55 to 95 °C. Relative telomere length (*T*/*R* ratio) was calculated by comparing the values of telomere (T) and reference gene *Alu* (R) in individual cells by the 2^−ΔΔCt^ method when the standard curves of telomere and *Alu* had similar high amplification efficiencies. A total of 302 cells were used for telomere length measurement and 242 cells passed RNA‐seq and telomere length QC (quality control) simultaneously (Table S8, Supporting Information). Primers for telomere and *Alu* were synthesized by IDT. Sample preparations were done in a special PCR hood.

##### 10 × Library Preparation and Sequencing

10 × library preparation was provided by Yale Center for Genome Analysis. scRNA‐seq libraries were generated using the Chromium Single Cell 3ʹ Reagent Kit v1 (patient 1)/v2 (patients 2, 3) (10 × Genomics) according to the manufacturer's protocol. Briefly, the single‐cell suspension in PBS with 0.04% BSA was mixed with RT‐PCR master mix and loaded together with Single Cell 3ʹ Gel Beads and Partitioning Oil into a Single Cell 3ʹ Chip according to the manufacturer's instructions. RNA transcripts from single cells were uniquely barcoded and reverse‐transcribed. cDNA molecules were preamplified, fragmented, end repaired and ligated with Illumina adapters to generate a single multiplexed library. All libraries were quantified by Qubit and 2100 export_High Sensitivity DNA Assay. Libraries were sequenced on HiSeq 2500, sequencing parameters were set according to the manufacturer's instructions.

##### Single‐Cell RNA Sequencing Data Analysis

Raw sequencing files were separated using their unique index combinations and to generate the FASTQ files. The human genome (GRCh38) and gene annotation file in GTF format were downloaded from Ensembl database (ftp://ftp.ensembl.org/pub/release02010;86/fasta/homo_sapiens/dna/Homo_sapiens.GRCh38.dna_sm.primary_assembly.fa.gz; ftp://ftp.ensembl.org/pub/release02010;86/gtf/homo_sapiens/Homo_sapiens.GRCh38.86.gtf.gz). Sequences and annotation of 92 “spike‐in” (from the External RNA Controls Consortium) were merged into human genome and its annotation file, respectively. scRNA‐seq data cleaning and quality control were conducted using the software Fastq_clean^[^ ^64^ ^]^ that is optimized to clean raw reads from Illumina platforms. Low quality (<Q20) bases on both ends of reads were trimmed and reads containing more than two ambiguous nucleotides (“N”) were removed. Adapter segments, template switch oligo (TSO) and Poly (A)^+^ tail sequences were trimmed. Subsequently, the cleaned paired‐end reads were aligned to the GRCh38 human reference genome using STAR aligner^[^ ^65^ ^]^ (version 2.5.2a) with options “–sjdbOverhang” set to “125,” “–outFilterMultimapNmax” set to “20,” “–alignMatesGapMax” set to “20000,” “–quantMode” set to “GeneCounts,” “–twopassMode” set to “Basic,” “–outReadsUnmapped” set to “None,” “–outFilterIntronMotifs” set to “RemoveNoncanonical” and “–quantMode” set to “GeneCounts” which results in the quantification of raw read counts at the gene level.

##### Selection of High‐Quality Transcriptomes from the Human CRC scRNA‐seq Experiment

R studio (https://www.rstudio.com/) was used to run in house R scripts to perform hierarchical clustering and PCA. To identify the distinct cell populations present in primary CRCs, cell clustering was performed using the R software package Seurat.^[^ ^19^ ^]^ The count matrix (total of 831 single cells) was first normalized by library size and log transformed by Seurat. Transcriptomes with fewer than 200 expressed genes and lowly expressed in three cells were discarded, cells with mitochondrial genes occupying more than 70% reads were defined as low‐quality cells and filtered out, this resulted in retention of 693 cells with 17565 genes for subsequent analysis.

##### Dimensionality Reduction Using PCA and Graph Clustering

The highly variable genes were identified by the set of genes that were most variable across single‐cell datasets using Seurat with default parameters. Dimensionality reduction was performed using principal component analysis (PCA), and statistically significant PCs were identified using the Jackstraw function in Seurat.^[^ ^19^ ^]^ Twenty significant PCs were identified for scRNA‐seq data. The scores of cells along these significant PCs were used to build a k‐nearest neighbor graph, and partition the cells into transcriptionally distinct clusters using the smart local moving community detection algorithm as implemented in the FindClusters function in Seurat. Subsequently, t‐distributed stochastic neighbor embedding (t‐SNE) was used to embed the cells based on statistically significant PCs, and to visualize the graph clustering output on a 2D map.

##### Identifying Markers for Each Cluster and Defining Distinct Subpopulations

To identify genes whose expression values could individually serve as a classifier for each cluster, Seurat's function “FindAllMarkers” was used^[^ ^19^ ^]^ (using recommended ROC test algorithms) to perform a differential expression analysis between the cells in the cluster of interest and the rest of the cells in the dataset. Marker specificity and precision was quantified using a statistical test based on the area under the precision‐recall curve (AUC) corresponding to the “classification power.” AUC is a quantitative measure of the balance between recall (the sensitivity of marker gene detection within the cluster of interest) and precision (accuracy of the quantitative levels of gene as a predictor of the correct cell type). Markers found by the analysis were “digital” (expressed only in the marked cluster) with AUC values > 0.7 (the AUC values ranged from 0 for random to 1 for perfect classifier for a given cluster). The AUC values of marker and non‐marker genes were compared at a range of expression values and show that the marker genes have significantly higher AUC values compared to non‐marker genes. Markers with low AUC values belong to smaller clusters in which a small number of false positives in other clusters can significantly reduce the AUC value. All markers identified by Seurat are provided in Table S2 (Supporting Information).

To define cell types from the t‐SNE cluster analysis, the CTen platform was used^[^ ^66^ ^]^ according to the highly expressed, cell‐specific (HECS) gene expression database to assess cell‐type specific enrichment results. Genes with AUC > 0.9 in each cluster for cell type enrichment analysis. The CTen enrichment score (‐log10 Benjamini‐Hochberg adjusted *P*‐value) of 2 or greater was considered significant. T cell, B cell, EPC, macrophage, dendritic cell and mast cell were directly identified by CTen platform. CSC was defined by the highly‐expressed genes and GO enrichment terms (i.e., term of “pluripotency of stem cells”). To ensure the specificity of the assignment of individual cells to each subpopulation, these identified clusters were further validated by their key marker genes. A full list of the genes preferentially expressed and reported as marker genes for each subpopulation is given in Table S3 (Supporting Information).

##### 10 × Genomics Computational Analysis

The Cell Ranger software suite was obtained from 10 × Genomics. Raw sequencing data was de‐multiplexed by Illumina bcl2fastq software to generate separate paired‐end read files for each sample, which were quality‐checked using FastQC software. The Cell Ranger “count” script was used to align human fastq files to the human GRCh38 reference genome (Ensembl). The raw count matrices were imported into R for further processing.

R studio (https://www.rstudio.com/) was used to run R scripts to perform hierarchical clustering and PCA. To identify distinct cell populations in primary CRC and normal tissues, cell clustering was performed using R software package Seurat 3.0.^[^ ^19^ ^]^ The count matrix was first normalized by library size and log transformed by Seurat. Transcriptomes with fewer than 200 expressed genes and lowly expressed in three cells were discarded, cells with mitochondrial genes occupying more than 40% (patient 1) or 70% (patients 2 and 3) of reads were defined as low‐quality cells and filtered out, this resulted in the retention of 3681 tumor cells and 6735 normal cells for subsequent analysis.

Six datasets of three patients’ tumor and normal cells were integrated by “IntegratedData” function of Seurat according to instructions. t‐distributed stochastic neighbor embedding (t‐SNE) was used for visualization and clustering. The “FindConservedMarkers” function was used to identify canonical cell‐type marker genes that are conserved across conditions. CTen^[^ ^66^ ^]^ and CellMarker^[^ ^67^ ^]^ platforms were used to define cell types from the t‐SNE cluster analysis.

##### Gene Set Enrichment Analysis

GO and KEGG enrichment analyses were performed using the *clusterProfiler* R package^[^ ^68^ ^]^ for DEGs of a distinct subpopulation based on the average fold change compared with other subpopulations. Enrichment scores were calculated by GSEA.^[^ ^69^ ^]^ In generalized GSEA, a gene set is considered enriched if the statistical significance (*P*‐value < 0.05) of its enrichment score is below the threshold. Network enrichment analysis was performed using Metascape (http://metascape.org).

##### Stemness, Telomerase Activity, and CSC Specific Signature Scores

Gene sets reflecting the expression signature of the “stemness” (including 63 genes)^[^ ^9^ ^]^ and “telomerase” (including 43 genes)^[^ ^14a^ ^]^ were identified in previous reports and validated. The telomerase score signature was derived from an unsupervised comparison of telomerase positive and negative cells, and the resulting genes were further narrowed by overlapping with genes overexpressed in embryonic stem cells.^[^ ^14a^ ^]^

##### Pseudotemporal Analysis of the Cell Lineage Trajectory

Single‐cell scaled expression data were analyzed with Monocle as previously described.^[^ ^34^ ^]^ Briefly, scaled data derived from Seurat were input into Monocle, which orders single cells according to subpopulations using an unsupervised algorithm method. Each cell can be viewed as a point in a high‐dimensional state space, and Monocle reconstructs the trajectory cells according to pseudotime. Hierarchical clustering analysis was performed for differentially expressed genes using expression values from all ordered cells.

##### Copy Number Inference from Single Cell RNA‐seq Data

Initial CNVs for each region were estimated by inferCNV R package (https://github.com/broadinstitute/inferCNV).^[^ ^1b^ ^]^ Raw count data were extracted from the Seurat object by “GetAssayData()” function. For the inferCNV analysis the following parameters were used: “denoise,” default hidden Markov model (HMM) settings, and a value of 0.1 for “cutoff.” The chromosomal expression patterns were estimated from the moving averages of 101 genes as the window size and adjusted as centered values across genes.

##### Cell Type Signature of TCGA Bulk expression Profiles of CRCs

Bulk CRC TCGA (The Cancer Genome Atlas) RNA‐seq data (htseq_fpkm‐uq) was downloaded from UCSC Xena website (http://xena.ucsc.edu/) and log2(fpkm‐uq+1)‐based gene quantifications along with additional tumor and clinical annotations. To further examine the classification of TCGA samples, all genes were identified preferentially expressed in each of the 11‐subtypes and scored single cells by the 11‐subtype gene‐sets. For each cell type identified in this study, cell type‐specific genes are defined as those: 1) with an AUC value above 0.7; and 2) *P* < 0.001 when comparing cells classified into that cell type to those in each other cell type.

##### Survival Analysis

Kaplan–Meier log‐rank tests were performed using default parameters in *SurvExpress*,^[^ ^38^ ^]^ an online biomarker validation tool and database. The tool takes gene lists as input, and in this case, differentially expressed genes with AUC>0.8 were used in each subpopulation. There are 22 Colon databases available, and the “COADREAD‐TCGA Colon and Rectum adenocarcinoma June 2016” dataset was selected that included 467 samples (http://bioinformatica.mty.itesm.mx:8080/Biomatec/SurvivaX.jsp). Survival analysis and risk assessment of the human CRC data set were applied. The samples were divided into two groups according to the average expression level of the genes in the gene list.

##### Cell Cycle Analysis

Cell cycle analysis was performed according to the previously described method.^[^ ^9^ ^]^ Briefly, average relative gene expression of a core set of 43 G1/S and 55 G2/M genes from the corresponding expression clusters in several previous studies were used to derive cell cycle scores (Table S5, Supporting Information). Scatter plot distribution of all cells along G1/S score and G2/M score revealed an approximate circle. The putative state of cycling cells was defined based on a previous description.^[^ ^9^ ^]^

##### Immunofluorescence Microscopy

After being deparaffinized, rehydrated and washed in PBS, paraffin sections were incubated with 3% H_2_O_2_ for 10 min at room temperature, subjected to high pressure antigen recovery sequentially in 0.01 m citrate buffer for 3 min, permeabilized in 0.1% Triton X‐100 for 30 min, blocked with 5% goat serum with 0.1% BSA in PBS for 2 h at room temperature, and then incubated with the primary antibodies against MKI67 (AB9260, Millipore), PCNA (sc‐25280, Santa Cruz), E‐CADHERIN (20874‐1‐AP, Proteintech), TNFRSF19 (sc‐398526, Santa Cruz), BAMBI (sc‐100681, Santa Cruz) KRT18 (A1022, ABclonal), CLDN4 (sc‐376643, Santa Cruz), CXADR (sc‐373791, Santa Cruz), or SLC12A2 (sc‐514858, Santa Cruz) overnight at 4 °C. This was followed by washing in PBS three times and incubation with secondary antibodies (Goat Anti‐Mouse IgG (H+L) FITC 115‐095‐003, Jackson; Goat Anti‐Rabbit IgG (H+L) Alexa Fluor 594 A‐11037, Life technology) for 2 h at room temperature. Blocking solution without the primary antibody served as a negative control. DAPI/Hoechst in Vectashield was used for staining of the nuclei. Fluorescence was imaged using a Zeiss LSM710 confocal microscope.

##### Immunofluorescence‐Telomere FISH

Sections for immunofluorescence were treated as described above. After incubation with secondary antibody, sections were rinsed in PBS three times for 5 min, fixed with 2% paraformaldehyde for 2 min, rinsed again, dipped through an ethanol series (70%, 95%, 100%), and air‐dried. Samples were denatured at 80 °C for 3 min and hybridized with the telomere PNA probe (0.5 µg mL^−1^, F1002, Panagene) in the dark for 2 h. Sections were washed in washing buffer (50% formamide, A100314‐0500; 10 × 10^−3^ m Tris–HCL PH7.2, T2069‐100ML, SIGMA) twice for 15 min, rinsed in PBS three times for 5 min and dried. Hoechst in Vectashield was used for nucleus staining.

##### Gene Knockout by CRISPR/Cas9

pSpCas9(BB)‐2A‐Puro (PX459) was a gift from Feng Zhang (Addgene plasmid # 48139). Guide RNAs were designed using the online design tool available at http://crispr.genome-engineering.org/. PX459 was digested with *Bbs*I and then gel purified. Two pairs of oligos including the target sequences were annealed and cloned into *Bbs*I‐digested PX459 vector. Primers used for CRISPR/Cas9 experiments are listed in Table S9 (Supporting Information).

##### Gene Expression by Quantitative Real‐Time PCR

Total RNA was isolated from cells using RNeasy mini kit (Qiagen). Two micrograms of RNA was subjected to cDNA synthesis using M‐MLV Reverse Transcriptase (Invitrogen). Real‐time quantitative PCR reactions were set up in duplicate with the FastStart Universal SYBR Green Master (Roche) and run on the iCycler iQ5 2.0 Standard Edition Optical System (Bio‐Rad) using primers detailed in Table S9 (Supporting Information). Each sample was repeated 3 times and analyzed using *ACTIN* as an internal control by comparative Ct (cycle threshold) method.

##### Analyses of Apoptosis by Flow Cytometry

Annexin V‐FITC antibody immunofluorescence was used to perform a fluorescent analysis of apoptosis and necrotic cells. Cells were harvested, centrifuged at 1000 rpm for 5 min, and washed two times with precooled PBS buffer. 1 × 10^5^ cells were collected and incubated with Annexin V‐FITC in the provided binding buffer for 20 min in dark at 4 °C, according to the instruction of the Annexin V‐FITC apoptosis detection Kit (C1063, Beyotime). They were then subjected to flow cytometer (FACS Calibur, BD) analysis at 488 nm of emission.

##### Data Presentation

Correlation plots, box plots, scatter plots, violin plots, bar graphs, and histograms were generated using the *ggplot2* R package (version 2.2.1). Heatmaps were generated using R package *pheatmap* (version 1.08), which were both obtained from CRAN (http://cran.r-project.org/).

##### Statistical Analysis

The data and number from multiple groups were analyzed by one‐way ANOVA. Pearson's linear correlation was used to test the correlation. Significant differences were defined as *P* < 0.05.

##### Ethics Approval and Consent to Participate

This study was approved by the Ethical Committee at Tianjin Medical University General Hospital (Ethical NO. IRB2014‐YX‐038), and informed consent was obtained prior to investigation. For 10 × Genomics, all patients were de‐identified and the standard operation procedures were approved by the Management Committee at Department of Pathology, Yale University School of Medicine (YSOP#100).

## Conflict of Interest

The authors declare no conflict of interest.

## Data Availability

Raw fastq files for single‐cell sequencing from this study have been submitted to the Sequence Read Archive (SRA; https://www.ncbi.nlm.nih.gov/sra) under accession number **SRP113436**. The custom Perl and R scripts used in this study are available upon request to the corresponding authors.

## Supporting information

Supporting Information

Supplemental Table 1
