# Supplementary material for: Colorectal Cancer Stem Cell States Uncovered by Simultaneous Single‐Cell Analysis of Transcriptome and Telomeres
Source: Adv Sci (Weinh). 2021 Feb 8;8(8):2004320. doi: 10.1002/advs.202004320 (PMC8061397; doi:10.1002/advs.202004320)
Supplement: Supplementary file 1 — Supporting Information [file ADVS-8-2004320-s001.pdf]

© 2020 Wiley-VCH GmbH

ADVANCED SCIENCE
Open Access

Supporting Information

for *Adv. Sci.*, DOI: 10.1002/advs.202004320

Colorectal cancer stem cell states uncovered by simultaneous single-cell analysis of transcriptome and telomeres

Hua Wang, Peng Gong, Tong Chen, Shan Gao, Zhenfeng Wu, Xiaodong Wang, Jie Li, Sadie L. Marjani, José Costa, Sherman M. Weissman, Feng Qi,* Xinghua Pan,* and Lin Liu**

Supporting Information

Colorectal cancer stem cell states uncovered by simultaneous single-cell analysis of transcriptome and telomeres

Wang *et al.*,

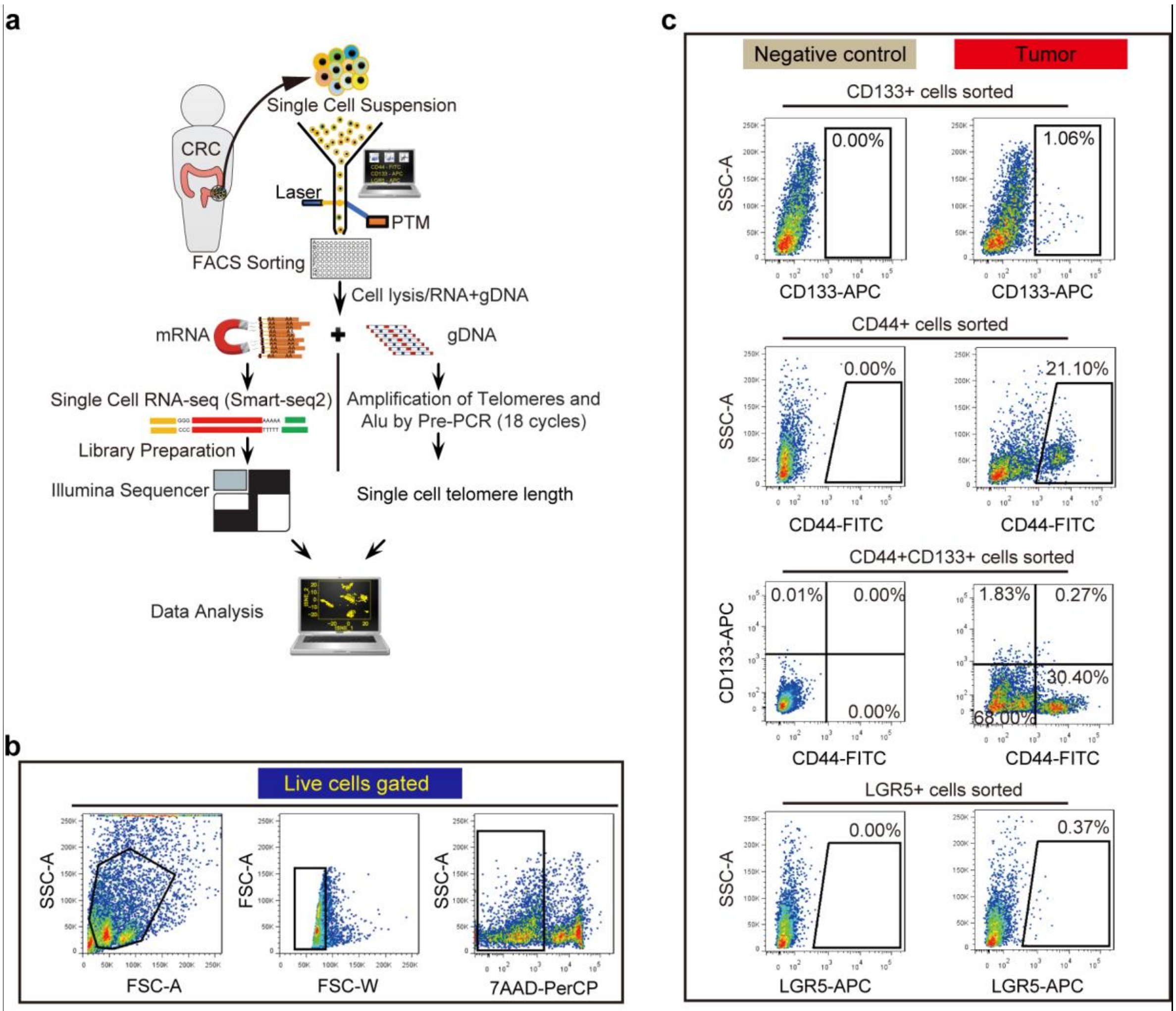

d

	Adhere	Sphere	LGR5+	LGR5-	CD44+	CD44-	CD133+	CD133-	CD44+ CD133+	CD44- CD133+	CD44- CD133-
CSCs	0	0	1	1	0	0	0	0	15	10	0
EPCs	2	10	22	2	11	2	53	7	9	1	0
EDCs	8	26	0	7	7	13	0	3	6	4	2
Ms	0	2	78	0	4	0	1	0	0	0	0
DCs	1	2	38	0	1	0	0	0	1	0	0
MSCs	1	30	17	3	1	0	0	2	7	15	6
TCs	5	1	2	12	55	24	2	3	3	0	0
BCs	17	0	5	21	51	3	11	9	0	0	0
MCs	0	0	0	0	28	0	0	0	0	0	0
CAFs	1	0	0	1	3	1	0	1	2	0	0
Total	35	71	163	47	161	43	67	25	43	30	8

Figure S1. Overview of workflow and enrichment of cancer stem cells by FACS sorting of CRC cells.

(**a**) Workflow overview. Tumor tissues were enzymatically digested, with red blood cells (RBC) removed by RBC lysis buffer, then the cell pellets were re-suspended in 0.1% BSA/PBS. The cell suspension was applied to FACS for sorting with known cell surface antibodies to mark CSCs. Each single cell was subjected to RNA-sequencing and telomere length measurement side-by-side at single-cell level. Tumors from eight patients were analyzed and sorted by FACS or isolated from cultured spheres. A total of 831 single cells were profiled by SMART-seq2, and among these cells, 302 cells were successfully sequenced and measured for telomere length simultaneously.

(**b**) Representative flow cytometry gating for live single cells. Successful gating showed

sequential selection of non-debris cells by size, and single cells gated as FSC-A and FSC-W. Dead cells were excluded by 7-AAD labeling.
(**c**) Representative flow cytometry gating for single-cell analysis by FACS. Negative control: no antibody added to the samples.
(**d**) Identification of the cells with message from SMART-seq2 for the cells following CSC enrichment by FACS. In addition to CSC enrichment by FACS using recognized CSC markers, cells from randomly selected (Adhere) and after sphere formation (Sphere) were also used for CSC enrichment.

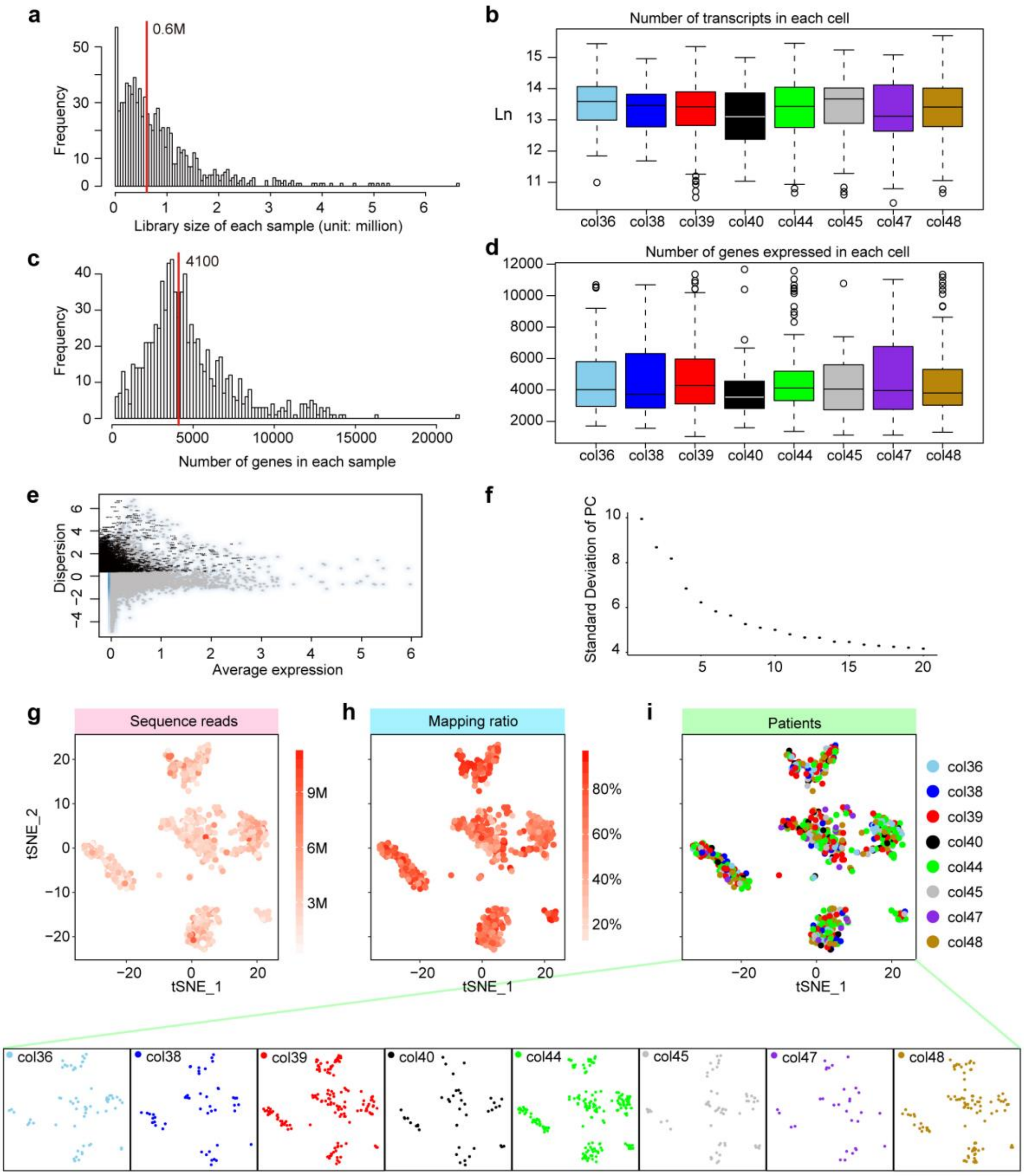

Figure S2. Quality controls of single-cell RNA-seq using SMART-seq2 and identification of variable genes across single cells.

(**a**) Histogram of the total number of reads (library size) per cell divided by the total number of sequenced transcripts as being counted with unique molecular identifiers (UMIs). The median of library size (red line) is 0.6 M.

(**b**) Boxplot of the number of transcripts detected in single cell.

(**c**) Histogram of the number of sequenced transcripts per cell. Mean number of detected genes (red line) is 4100. The 831 cells were sequenced on 12 lanes to a total depth of 2,185,235,284 paired reads.

(**d**) Boxplot of the number of genes detected in single cell.

(**e**) Identification of the highly variable genes for principle component analysis (PCA) and t-distributed stochastic neighbor embedding (t-SNE analyses) across all cells passed QC by *Seurat*. Each point is the dispersion estimated for each gene obtained by *Seurat*. Black points indicate highly variable genes that show high gene dispersion

estimation.
(**f**) Plot of the standard deviations of the principle components. The ‘ad hoc’ method of *Seurat* for determining cutoff to use is to look at a plot of the standard deviations of the principle components and to draw the cutoff where there is a clear elbow in the graph. It appears that the elbow falls around principle component 17.
(**g**) t-SNE plot of total clean sequence reads in single cell.
(**h**) t-SNE plot of mapping ratio in single cell.
(**i**) t-SNE plot of cells from different patients. Bottom panel, patient samples are broken apart into individual plots, showing that all clusters are present in all patients.

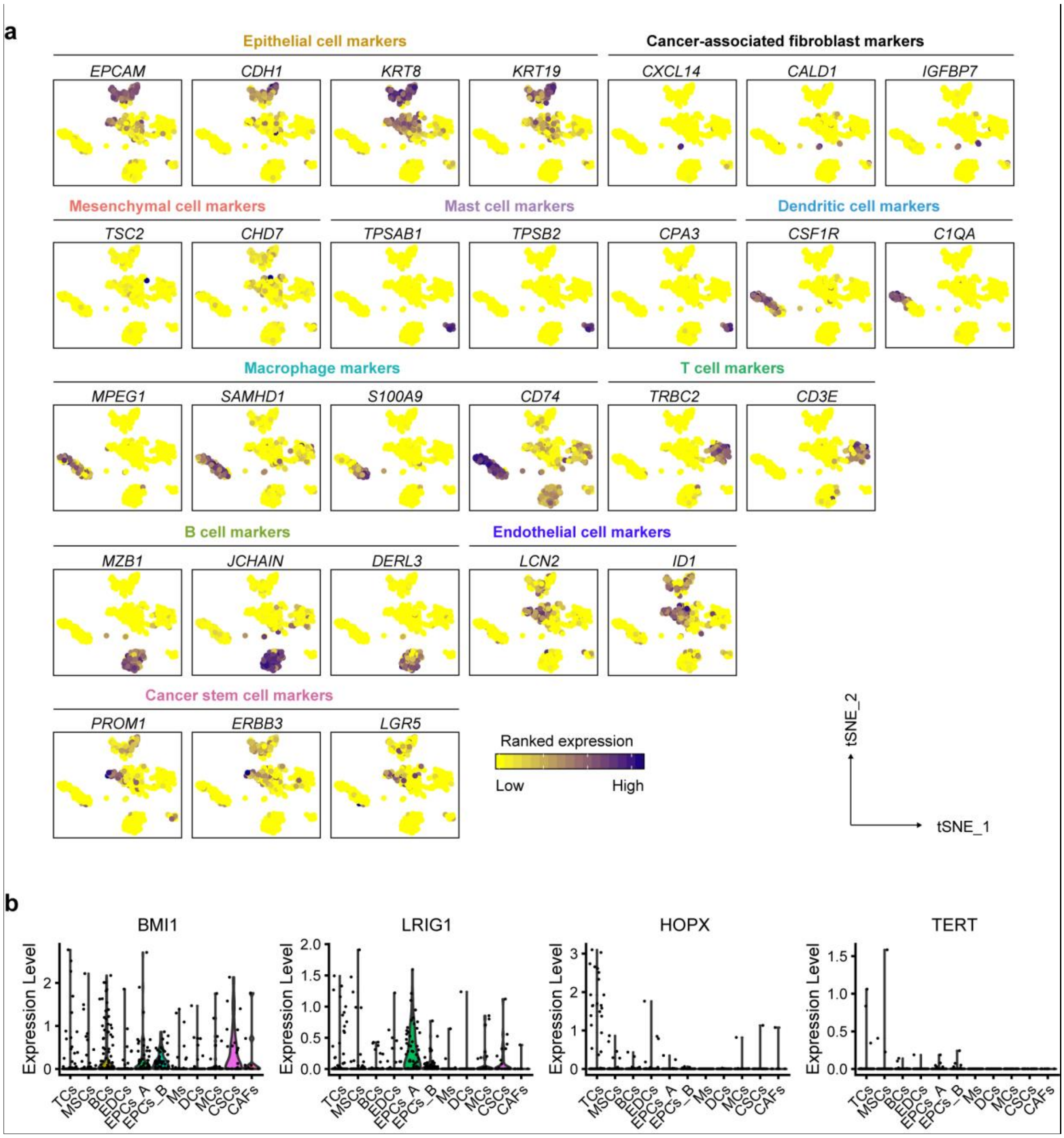

Figure S3. Expression patterns of marker genes exhibited on t-SNE plots.

(**a**) Decomposing on t-SNE plot the distribution of distinct markers expressed for each cluster. Color key represents expression level of marker genes by a gradient of yellow and navy indicating lower to higher expression.

(**b**) Expression patterns of the marker genes of intestinal stem cells, *BMI1*, *HOPX*, *TERT* and *LRIG1*.

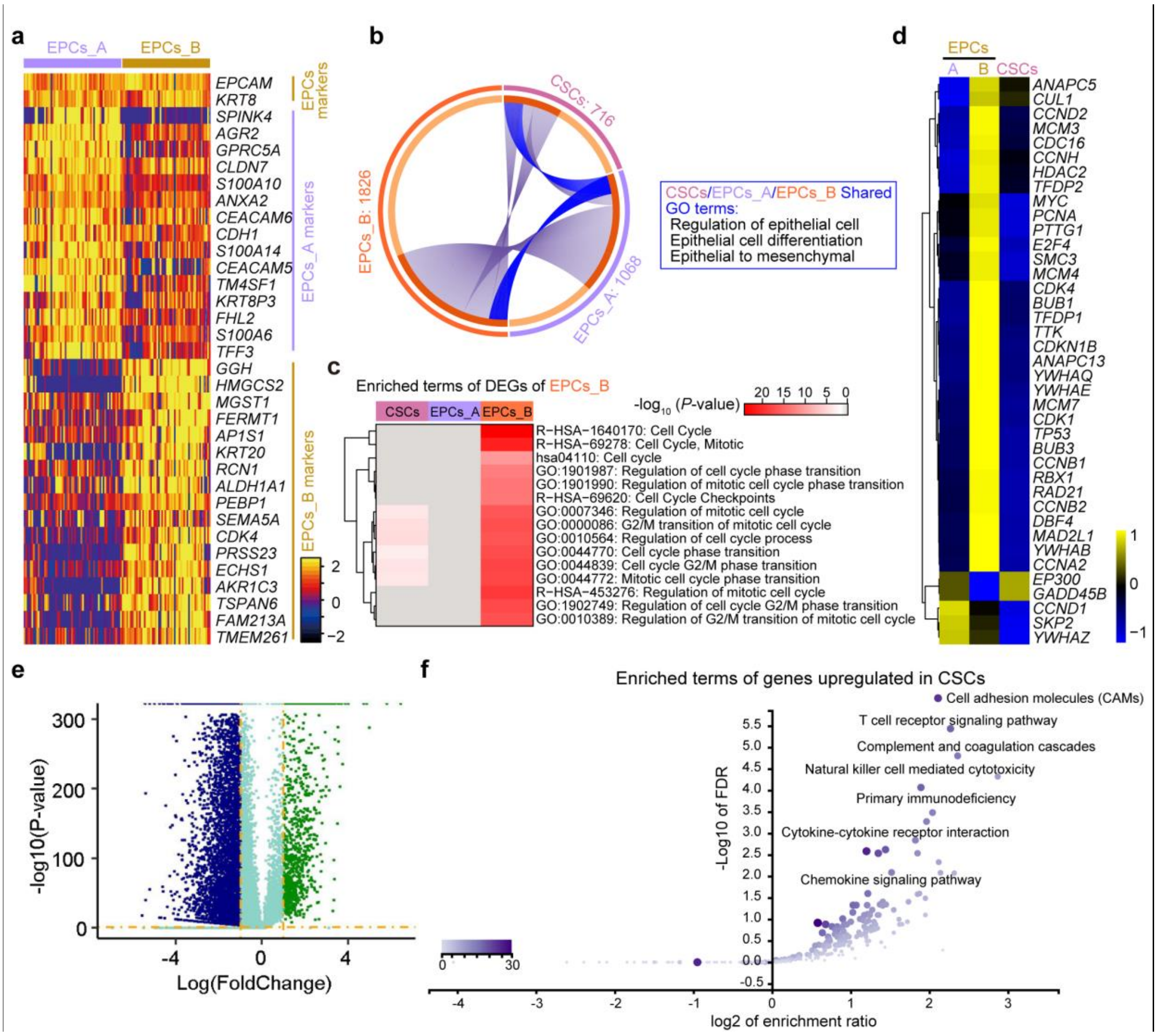

Figure S4. Comparison of the two subpopulations of cancer epithelial cells (EPCs) and CSCs.

(**a**) Heatmap showing the DEGs and shared genes in the two subpopulations of cancer EPCs.

(**b**) Circos plot (Tripathi et al., 2015) showing interaction between EPCs and CSCs. The shared genes in the two cell types are linked by purple lines, and the shared genes in all three cell types are linked by blue lines. Right, shared genes among EPCs and CSCs are enriched gene ontology terms related to the functional of epithelial cells.

(**c**) Heatmap showing the enrichment of DEGs of EPCs_B versus CSCs and EPCs_A. The color key from white to red indicates higher to lower *P*-values, respectively.

(**d**) Heatmap indicating hierarchical clustering of average gene signatures involving cell-cycle regulation.

(e) Volcano plot displaying DEGs between CSCs and EPCs. Genes are plotted as log2 fold change versus the -log10 of the adjusted p-value. Genes in orange denote those upregulated in CSCs while navy denotes upregulated in EPCs. Thresholds are shown as dashed lines.

(**f**) Volcano plot showing enrichment of DEGs of CSCs compared with EPCs. The size of the dots represents the number of genes in the significant DE gene list associated with the term and the color of the dots represent the –log10 of *P*-values.

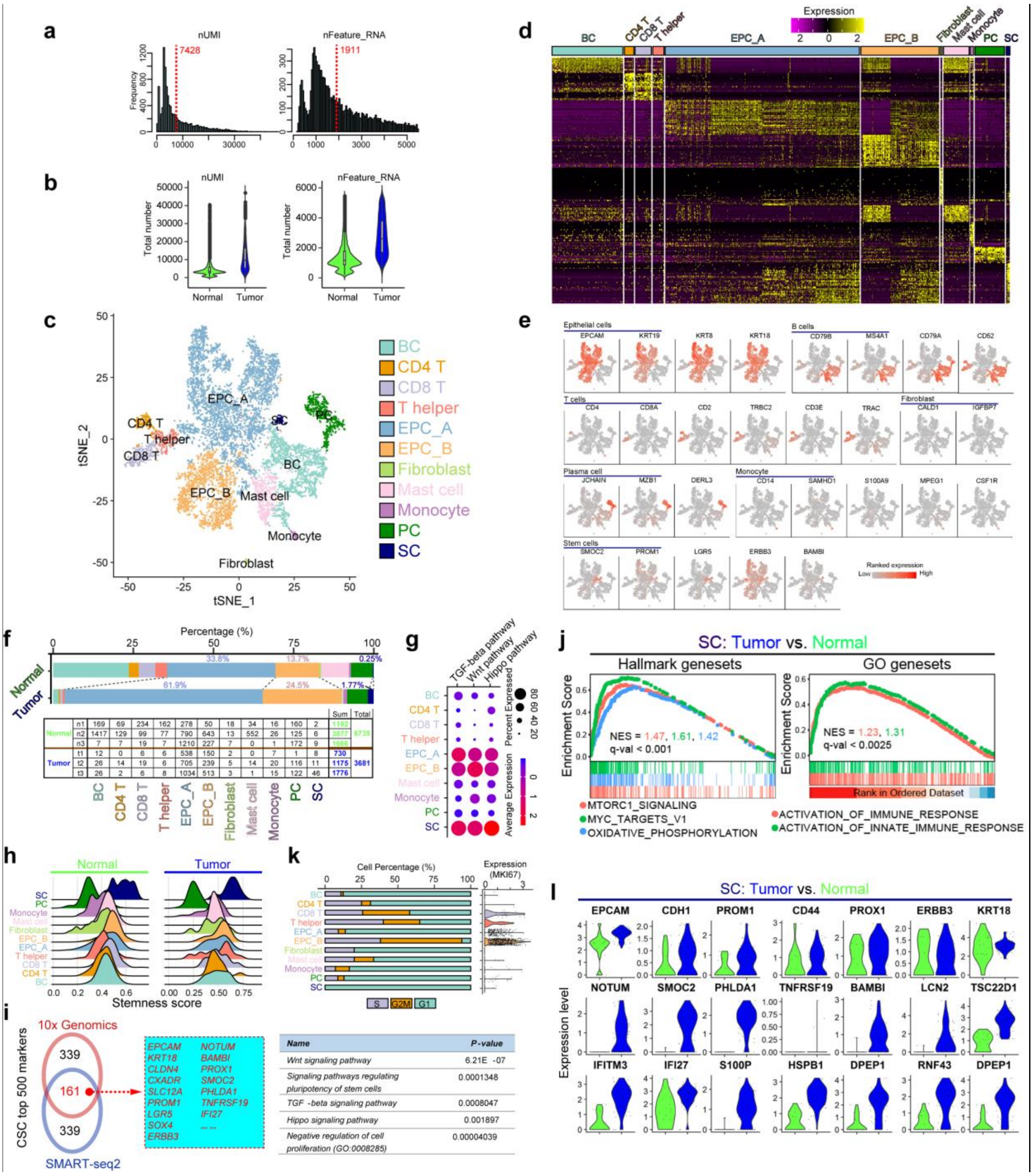

Figure S5. Characterization of CSCs and EPCs by scRNA-seq analyzed on 10× Genomics.

(**a**) Distribution of UMIs (Left) and number of genes (Right) detected per cell for the cells from CRCs and healthy control tissues. The average number is indicated by red dashed line.

(**b**) Violin plots of the number of UMIs (Left) and expressed genes (Right) detected per cell for the cells from CRCs and healthy control tissues. Boxes represent the median and the first and third quartiles.

(**c**) t-SNE plot displaying distinct cell subpopulations from CRC and healthy control tissues. t-SNE plot was based on the high variations of expressed genes by *Seurat*. Each point represents an individual cell. Cell clusters were labeled by sub-cell type names according to their specific marker genes and colored according to the clusters identified.

(**d**) Heatmap showing single-cell expression levels for the top 50 most specific genes for

each cell cluster. Rows represent genes, and columns represent cells.
(**e**) t-SNE plots illustrating distribution of marker genes for each cell type. Color key represents expression level of cell type marker genes by a gradient from gray to red.
(**f**) Numbers of single cells of different cell types in CRCs and healthy controls across the patients.
(**g**) Dot plots of functionally relevant genes expression involved in WNT pathway, HIPPO pathway and TGF-β pathway. Dot size represents the fraction of gene expressing cells and color intensity represents gene expression levels.
(**h**) Density distribution of stemness score in subpopulations of all the cells, CRCs and the healthy controls.
(**i**) Overlap genes and enriched GO terms between our SMART-seq2 and 10x Genomics data of the top 500 genes in stem cell population.
(**j**) GSEA exhibiting the enrichment of KEGG pathways and Gene Ontology (GO) terms for the DEGs in stem cells between CRCs and healthy tissues. The curve lines represent the evolution of the density of the genes identified in the ordered gene list based on the fold changes. NES, normalized enrichment score; q-val (q value), adjusted p value for the false discovery rate (FDR).
(**k**) Proportion of cell cycle phases for different cell types in CRC based on the single cell RNA-seq data. Right, violin plot show MKI67 expression in the cells from different cell types. BC, B cell; T, T cell; EPC, epithelial cell; PC, plasma cell; SC, stem cell.
(**l**) Violin plot showing the representative similarly and differentially expressed genes in stem cells from CRC tumor and healthy controls.

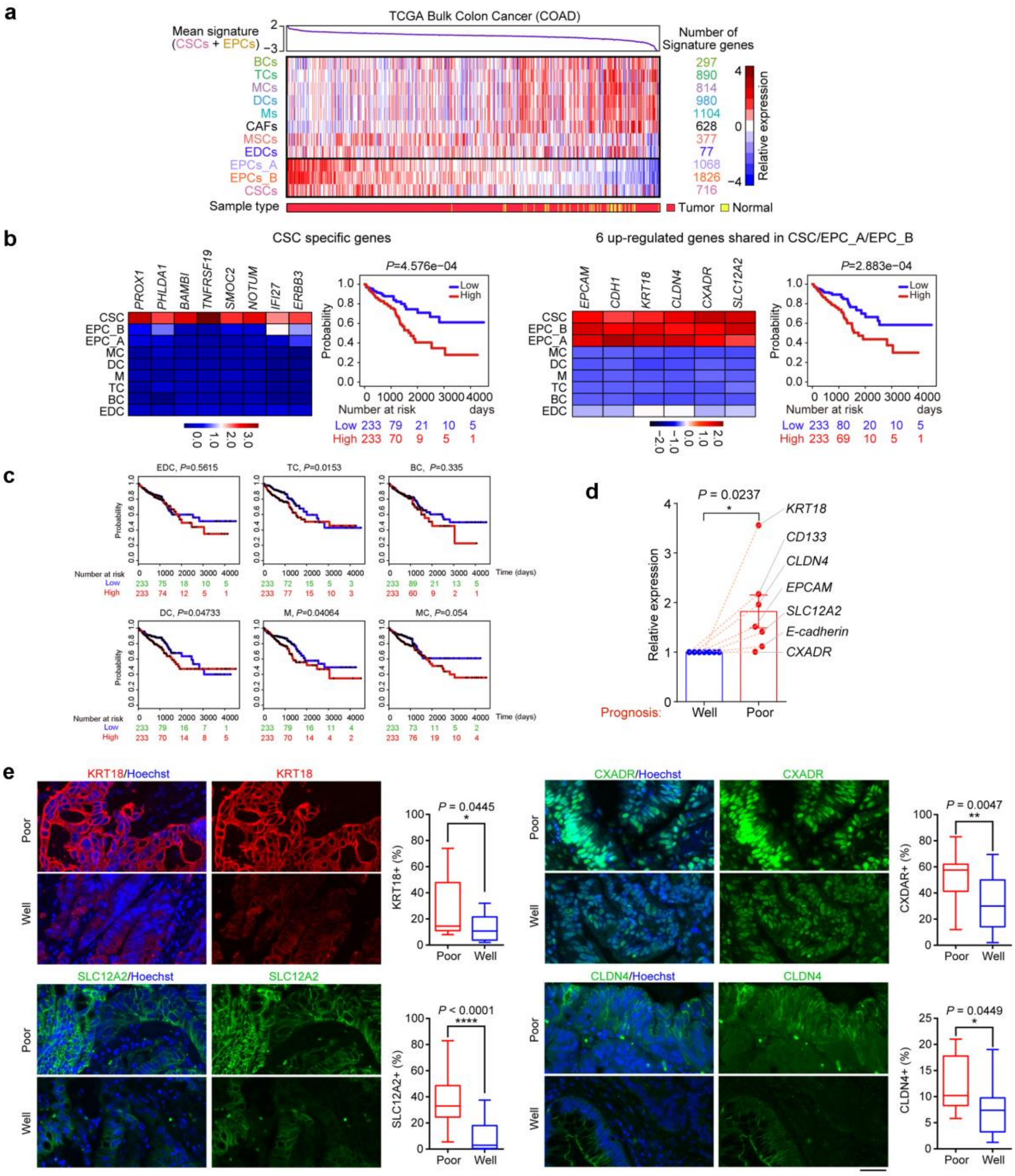

Figure S6. Prediction of prognosis by molecular signature of CSCs and EPCs.
(**a**) Bulk tumors were ordered on the basis of their inferred cell type composition defined from single cell RNA-seq. The top panel shows signature score of “Epithelial lineage”. Heatmap shows the relative average level of gene signatures of inferred cell types representing an index of cell type abundances of the CRC tissues (row) across 467 TCGA bulk-RNA signatures for CRC (column).
(**b**) Survival curve for 8 genes specific for CSCs and 8 genes highly-expressed in both CSCs and EPCs. Kaplan–Meier log-rank tests were performed using default parameters in *SurvExpress*, an online biomarker validation tool and database (http://bioinformatica.mty.itesm.mx:8080/Biomatec/SurvivaX.jsp). Statistical significance was assessed using a log-rank test.
(**c**) Kaplan-Meier survival analysis of cell subpopulations based on clinical data by the signature for other subpopulations beyond CSCs and EPCs.

(**d**) Prognosis analysis by qPCR of our clinical samples using seven marker genes shared by CSCs and EPCs. Three patients with recurrence (poor) and three patients without recurrence (well) at three years after surgery with data available are included. (**e**) Immunofluorescence of specific shared genes by CSCs and EPCs in tumors from poor and well prognosis patients. Right panel, Quantification of the corresponding antigen-positive tumor cells in poor and well prognosis patients. The poor prognosis samples here were defined as the patients with recurrence and/or death within three years, and the three patients-only available at the time of this data analysis were grouped into poor prognosis. Four control samples were randomly selected from the patients with no recurrence within three years. Bar graphs quantify the percentage of primary tumor cells that were positive for the antibody staining. The threshold in Adobe Photoshop was arbitrarily adjusted to exclude background staining. Quantification was estimated from at least four fields from each patient tumor, and at least 200 cells were randomly analyzed in each field. Data presented as mean $\pm$ SD. *, $P < 0.05$; **, $P < 0.01$; ****, $P < 0.0001$ by t-test. Scale bar = 20 μm.

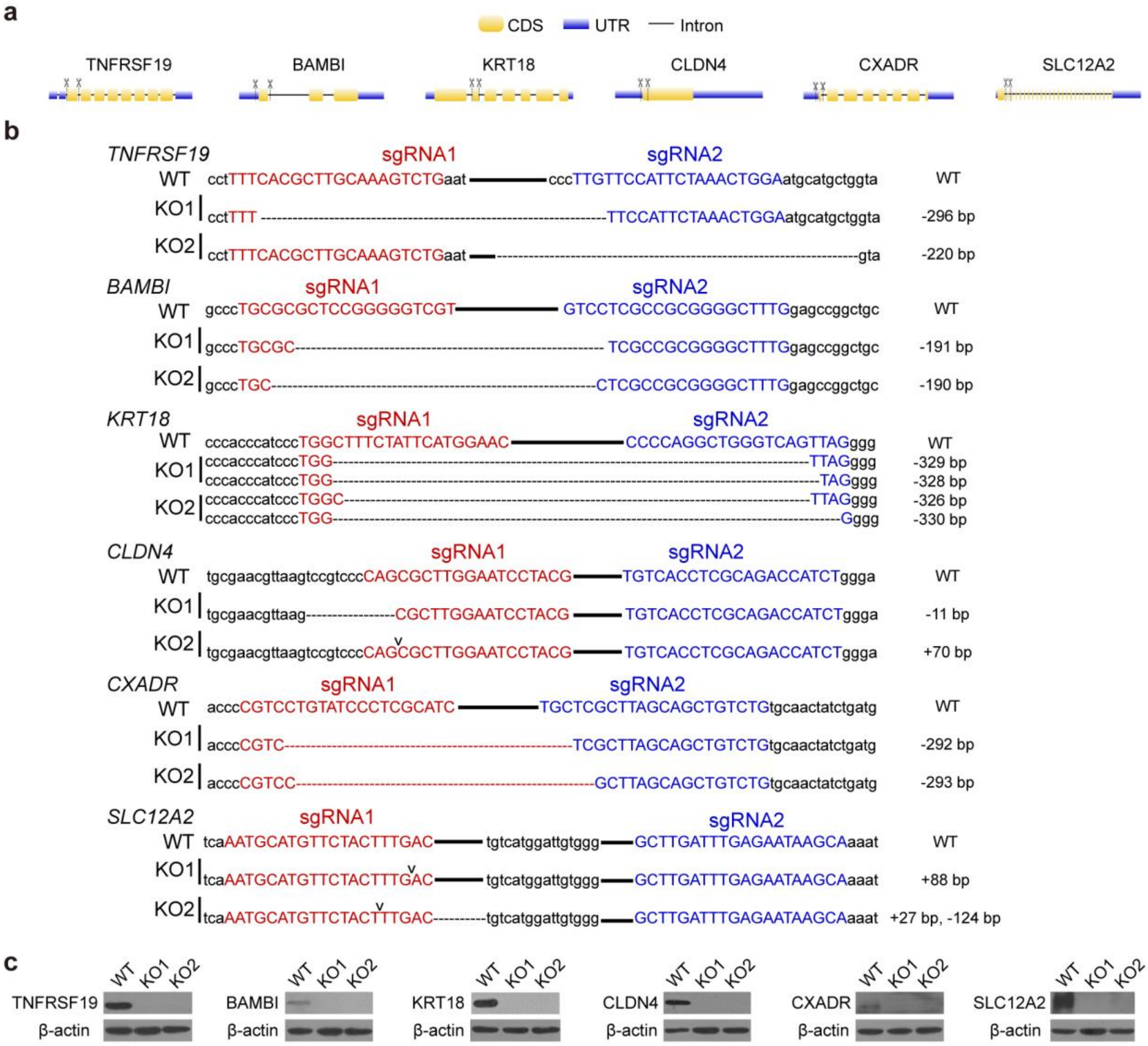

Figure S7. Targeted disruption of the specific enriched genes in human colorectal cancer cell HCT116 by CRISPR/Cas9.

(**a**) Scheme for knock-out of marker genes by CRISPR/Cas9 in HCT116. Two sgRNAs were used for construct knock-out cell lines; sgRNA-Left and sgRNA-Right of each genes are indicated by black imaginary lines. Cas9-GFP expression plasmids contained two sgRNAs were transfected into HCT116 by Lipo2000 system, and GFP positive cells were sorted by FACS at the 48h after transfection.

(**b**) Sequencing results of deletions or insertions induced by CRISPR/Cas9 in HCT116 clones. Deletions are indicated by dashes, and insertion indicated by downward arrows. The sgRNA1 recognition site is labeled in red and sgRNA2 in blue.

(**c**) Protein levels assayed by Western blot of the genes after knock-out at the passage 2. Two knock-out cell lines were selected and validated by sequencing the target genomic site.

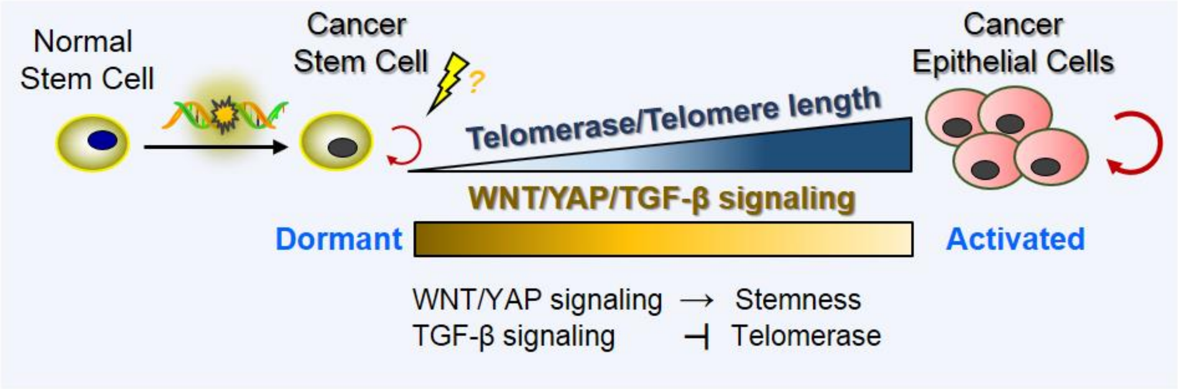

Figure S8. Simplified model of CSCs at dormant state and linkage to cancer epithelial cells (EPCs).

The model lists examples of stemness, telomere and telomerase, proliferation and representative singling pathways of CSCs and transition to EPCs. The levels in light color to dark color indicate minimal to high.

Tables S1-10:

Table S1. Clinical information of colorectal cancer patients included in scT&R-seq from Tianjin General Hospital

Table S2. List of genes specifically expressed in each subpopulation

Table S3. References listed for representative markers of cell subpopulations

Table S4. The enriched gene ontology terms (biological processes) showing the cell properties of cell types

Table S5. Gene sets used to define cell cycle, stemness and telomerase scores

Table S6. KEGG enrichment of upregulated genes in CSCs compared with other subpopulations

Table S7. GO analysis of DEGs from EPCs_A cells compared with EPCs_B cells

Table S8. Relative telomere length and cell cycle, stemness, and clusters

Table S9. List of primer sequences used in this study

Table S10. Clinical information of colorectal cancer patients included in 10× Genomics scRNA-seq
